# Global Role and Burden of Influenza in Pediatric Respiratory Hospitalizations, 1982–2012: A Systematic Analysis

**DOI:** 10.1371/journal.pmed.1001977

**Published:** 2016-03-24

**Authors:** Kathryn E. Lafond, Harish Nair, Mohammad Hafiz Rasooly, Fátima Valente, Robert Booy, Mahmudur Rahman, Paul Kitsutani, Hongjie Yu, Guiselle Guzman, Daouda Coulibaly, Julio Armero, Daddi Jima, Stephen R. C. Howie, William Ampofo, Ricardo Mena, Mandeep Chadha, Ondri Dwi Sampurno, Gideon O. Emukule, Zuridin Nurmatov, Andrew Corwin, Jean Michel Heraud, Daniel E. Noyola, Radu Cojocaru, Pagbajabyn Nymadawa, Amal Barakat, Adebayo Adedeji, Marta von Horoch, Remigio Olveda, Thierry Nyatanyi, Marietjie Venter, Vida Mmbaga, Malinee Chittaganpitch, Tran Hien Nguyen, Andros Theo, Melissa Whaley, Eduardo Azziz-Baumgartner, Joseph Bresee, Harry Campbell, Marc-Alain Widdowson, Global Respiratory Hospitalizations—Influenza Proportion Positive (GRIPP) Working Group

**Affiliations:** 1 Influenza Division, Centers for Disease Control and Prevention, Atlanta, Georgia, United States of America; 2 School of Health Sciences, University of Tampere, Tampere, Finland; 3 Centre for Global Health Research, University of Edinburgh, Edinburgh, United Kingdom; 4 Public Health Foundation of India, New Delhi, India; 5 Afghanistan National Public Health Institute, Ministry of Public Health, Kabul, Afghanistan; 6 National Directorate of Public Health, Ministry of Health, Luanda, Angola; 7 National Centre for Immunisation Research and Surveillance, The Children’s Hospital at Westmead, Westmead, New South Wales, Australia; 8 Institute of Epidemiology, Disease Control and Research, Dhaka, Bangladesh; 9 Division of Infectious Disease, Key Laboratory of Surveillance and Early-warning on Infectious Disease, Chinese Centre for Disease Control and Prevention, Beijing, China; 10 Caja Costarricense de Seguro Social, San José, Costa Rica; 11 Pasteur Institut of Côte d’Ivoire, Abidjan, Côte d’Ivoire; 12 Ministerio de Salud de El Salvador, San Salvador, El Salvador; 13 Ethiopian Public Health Institute, Addis Ababa, Ethiopia; 14 Medical Research Council Unit, Fajara, The Gambia; 15 Department of Paediatrics, University of Auckland, Auckland, New Zealand; 16 Centre for International Health, University of Otago, Dunedin, New Zealand; 17 Noguchi Memorial Institute for Medical Research, University of Ghana, Accra, Ghana; 18 Ministerio de Salud Publica y Asistencia Social, Guatemala City, Guatemala; 19 National Institute of Virology, Pune, India; 20 National Institute of Health Research and Development, Jakarta, Indonesia; 21 Centers for Disease Control and Prevention, Nairobi, Kenya; 22 Ministry of Health, Bishkek, Kyrgyzstan; 23 National Influenza Centre, Virology Unit, Institut Pasteur of Madagascar, Antananarivo, Madagascar; 24 Universidad Autónoma de San Luis Potosí, San Luis Potosí, Mexico; 25 National Centre for Public Health, Chisinau, Republic of Moldova; 26 National Influenza Center, Ulaanbaatar, Mongolia; 27 Institut National d’Hygiène, Ministère de la Santé, Rabat, Morocco; 28 Federal Ministry of Health, Abuja, Nigeria; 29 Ministerio de Salud Publica y Bienestar Social, Asunción, Paraguay; 30 Research Institute for Tropical Medicine, Manila, Philippines; 31 Ministry of Health, Kigali, Rwanda; 32 National Institute for Communicable Diseases, Johannesburg, South Africa; 33 Zoonoses Research Unit, Department Medical Virology, University of Pretoria, Pretoria, South Africa; 34 Division of Global Health Protection, Centers for Disease Control and Prevention, Atlanta, Georgia, United States of America; 35 Ministry of Health, Dar es Salaam, Tanzania; 36 National Institute of Health, Ministry of Public Health, Nonthaburi, Thailand; 37 National Institute of Hygiene and Epidemiology, Hanoi, Viet Nam; 38 Virology Laboratory, University Teaching Hospital, Lusaka, Zambia; Makerere University Medical School, UGANDA

## Abstract

**Background:**

The global burden of pediatric severe respiratory illness is substantial, and influenza viruses contribute to this burden. Systematic surveillance and testing for influenza among hospitalized children has expanded globally over the past decade. However, only a fraction of the data has been used to estimate influenza burden. In this analysis, we use surveillance data to provide an estimate of influenza-associated hospitalizations among children worldwide.

**Methods and Findings:**

We aggregated data from a systematic review (*n =* 108) and surveillance platforms (*n =* 37) to calculate a pooled estimate of the proportion of samples collected from children hospitalized with respiratory illnesses and positive for influenza by age group (<6 mo, <1 y, <2 y, <5 y, 5–17 y, and <18 y). We applied this proportion to global estimates of acute lower respiratory infection hospitalizations among children aged <1 y and <5 y, to obtain the number and per capita rate of influenza-associated hospitalizations by geographic region and socio-economic status.

Influenza was associated with 10% (95% CI 8%–11%) of respiratory hospitalizations in children <18 y worldwide, ranging from 5% (95% CI 3%–7%) among children <6 mo to 16% (95% CI 14%–20%) among children 5–17 y. On average, we estimated that influenza results in approximately 374,000 (95% CI 264,000 to 539,000) hospitalizations in children <1 y—of which 228,000 (95% CI 150,000 to 344,000) occur in children <6 mo—and 870,000 (95% CI 610,000 to 1,237,000) hospitalizations in children <5 y annually. Influenza-associated hospitalization rates were more than three times higher in developing countries than in industrialized countries (150/100,000 children/year versus 48/100,000). However, differences in hospitalization practices between settings are an important limitation in interpreting these findings.

**Conclusions:**

Influenza is an important contributor to respiratory hospitalizations among young children worldwide. Increasing influenza vaccination coverage among young children and pregnant women could reduce this burden and protect infants <6 mo.

## Abstract 2

The substantial global burden of influenza infections in children is revealed by Lafond and colleagues. Children in developing countries are 3 times more likely to be hospitalised and treatments vary. This study highlights the need for vaccination programs in the young.

## Editors' Summary

**Background:**

Acute lower respiratory infections—bacterial and viral infections of the lungs and airways (the tubes that take oxygen-rich air to the lungs)—are major causes of illness and death in children worldwide. Pneumonia (infection of the lungs) alone is responsible for 15% of deaths among children under five years old and kills nearly one million young children every year. Globally, infections with respiratory syncytial virus and with *Streptococcus pneumoniae* are associated with about 25% and 18.3%, respectively, of all episodes of severe respiratory infection in young children. Another infectious organism that contributes to the global burden of respiratory disease among children is the influenza virus. Every year, millions of people become infected with this virus, which infects the airways and causes symptoms that include a high temperature, tiredness and weakness, general aches and pains, and a dry chesty cough. Most infected individuals recover quickly, but seasonal influenza outbreaks (epidemics) nevertheless kill about half a million people annually, with the highest burden of severe disease being experienced by elderly people and by children under five years old.

**Why Was This Study Done?:**

Annual immunization (vaccination) can reduce an individual’s risk of catching influenza, but before a country implements this preventative measure, policymakers need reliable estimates of the burden of influenza in their country. Although such estimates have been calculated for resource-rich countries with temperate climates, where influenza largely occurs in the winter, few estimates of influenza burden are available for resource-limited countries, which has hampered informed consideration of vaccination for influenza prevention in many settings. Recently, however, there has been a global expansion of systematic surveillance and testing for influenza virus among patients admitted to hospital for severe respiratory infection. Here, the researchers use this expanded surveillance data to provide an estimate of influenza-associated hospitalizations among children worldwide between 1982 and 2012. Specifically, they undertake a systematic review to identify published research articles on influenza-associated respiratory disease in hospitalized children, and, by aggregating the data from these articles with data collected by hospital-based influenza surveillance, they calculate a pooled estimate of the proportion of children hospitalized with respiratory disease who are positive for influenza.

**What Did the Researchers Do and Find?:**

Using predefined search criteria, the researchers identified 108 published research articles that provided information on influenza-associated respiratory illness among hospitalized children. In addition, the Global Respiratory Hospitalizations–Influenza Proportion Positive (GRIPP) working group provided 37 hospital-based influenza surveillance datasets. By aggregating the data from these sources using a statistical approach called meta-analysis, the researchers calculated that, overall, influenza was associated with 9.5% of hospitalizations for severe respiratory infection among children under 18 years old worldwide, ranging from 4.8% among children under six months old to 16.4% among children aged 5–17 years. The researchers also calculated that, on average over the study period, influenza resulted in about 374,000 hospitalizations annually among children under one year old (including 228,000 hospitalizations among children less than six months old) and nearly one million hospitalizations annually among children under five years old. Finally, the researchers calculated that influenza-associated hospitalization rates among children under five years old over the study period were more than three times higher in resource-limited countries than in industrialized countries (150 and 48 hospitalizations, respectively, per 100,000 children per year).

**What Do These Findings Mean?:**

Differences in hospitalization practices, in applications of case definitions, and in influenza testing protocols between settings may affect the accuracy of these findings. Specifically, the approach taken by the researchers may mean that their estimate of the total burden of severe respiratory disease due to influenza is an underestimate of the true situation. Even so, these findings suggest that influenza is an important contributor to hospitalizations for severe respiratory illness among children worldwide. Increasing influenza vaccination coverage among young children and pregnant women could, therefore, reduce the contribution that influenza makes to hospitalizations for respiratory infections among children. Importantly, the estimates of the burden of influenza provided by these findings can now be used by countries considering influenza vaccination programs for children and/or pregnant women to help them investigate the possible health and cost implications of such programs and should also stimulate further research into the development of effective influenza vaccines for young children.

**Additional Information:**

This list of resources contains links that can be accessed when viewing the PDF on a device or via the online version of the article at http://dx.doi.org/10.1371/journal.pmed.1001977.

- The UK National Health Service Choices website provides information about respiratory infections, seasonal influenza, influenza vaccination, and influenza vaccination in children
- The World Health Organization provides information on seasonal influenza (in several languages) and on influenza vaccines
- The US Centers for Disease Control and Prevention also provides information for patients and health professionals on all aspects seasonal influenza, including information about vaccination, and about children, influenza, and vaccination; its website contains a short video about personal experiences of influenza
- Flu.gov, a US government website, provides access to information on seasonal influenza and vaccination
- MedlinePlus has links to further information about influenza and about vaccination (in English and Spanish)

## Introduction

Influenza virus infections are a substantial contributor to respiratory morbidity and mortality, with the highest burden of severe disease experienced by those aged <5 y and ≥65 y [1–3]. Until recently, however, estimates of influenza burden have been largely obtained from resource-rich settings with temperate climates with clearly defined influenza seasonality, with few estimates available from lower-income countries [4–6]. This lack of information on influenza burden in resource-limited settings has hampered informed consideration of implementation of preventive measures such as vaccination.

In the last 10 y, however, the global expansion of influenza surveillance and laboratory capacity for influenza testing by reverse transcription PCR has led to dramatic increases in testing in settings with previously sparse data. Many countries worldwide now perform hospital-based influenza surveillance among patients hospitalized with severe acute respiratory illness (SARI) [7–9]. These systems have proven useful in tracking influenza activity among hospitalized patients, but only a fraction of the collected data has been used to estimate the burden of influenza-associated hospitalizations. A 2011 meta-analysis using data from 16 population-based research sites and published literature estimated that there were 1 million cases of influenza-associated SARI episodes globally in children aged <5 y old in 2008. In this study, the per capita rate of severe influenza illness in developing countries was at least double that of industrialized countries [6]. This rate-based approach was mainly derived from sites with a limited population at risk under surveillance, and therefore represented only a small fraction of hospitals conducting surveillance for severe influenza disease globally.

We aimed to update the previous influenza burden estimates in young children, making full use of updated and expanded surveillance data from the past decade (2003–2012), both before and after the 2009 pandemic influenza emergence. We further aimed to extend previous studies by providing global estimates of the prevalence of influenza among acute lower respiratory infection (ALRI) hospitalizations among both younger (<5 y) and older (5–17 y) children.

## Methods

We aggregated data from all eligible published etiologic studies of influenza-associated respiratory illness among hospitalized children, which we supplemented with data from a working group of inpatient surveillance systems worldwide. We then calculated a final pooled estimate of the proportion of tested samples that were positive for influenza by reverse transcription PCR among children aged <18 y (referred to as proportion or percent positive), using age-group-specific random-effects log-binomial regression models. Finally, we applied the aggregate pooled proportion positive among children <1 and <5 y to age-specific denominators of global hospitalizations for ALRI among these two age groups [10] to obtain the number and rate (per 100,000 children per year) of pediatric influenza-associated hospitalizations, by World Health Organization (WHO) region and United Nations (UN) country development status.

### Systematic Review of the Literature

We searched nine online databases (PubMed, Embase, Web of Science, CINAHL [Cumulative Index to Nursing and Allied Health Literature], IndMed, LILACS [Literatura Latino-Americana e do Caribe em Ciências da Saúde], WHOLIS [WHO Library Database], CNKI [China National Knowledge Infrastructure], and the Global Health Database) to identify articles published from 1 January 1996 to 1 June 2012. The search was conducted with no language restrictions, and full search terms are provided in S1 Table. Briefly, keywords included “influenza” or “viral etiology” and other designators of respiratory illness such as “acute respiratory infection” and “influenza-like illness”. Searches via the CNKI Chinese-language database were conducted by native Mandarin speakers.

Identified articles were screened by two independent reviewers (two from K. E. L., M. W., E. A.-B., M.-A. W., P. Glew, S. Mei, Z. Suizan) for inclusion in the analysis, and duplicates were removed. The inclusion criteria were as follows: (1) original study with human participants, (2) laboratory testing for influenza, with description of the type of diagnostic method used, (3) minimum of 12 mo of continuous surveillance, (4) specified case definition (such as ALRI, SARI, or acute respiratory illness) or other clear criteria for specimen collection and testing, (5) hospitalized patients (excluding nosocomial infections), (6) number of enrolled cases from whom clinical specimens were collected and found positive was provided, and (7) minimum of 50 children (<18 y or “pediatric” as defined by authors) tested for influenza, in order to screen out small, potentially unreliable studies from the study dataset. For title and abstracts that met these criteria, full-text articles were obtained and re-screened. Full-text articles written in languages other than English were screened twice by co-investigators who could read the relevant language. Any discrepancies were discussed and resolved by reviewers. Independent screening was concordant for 92% of full-text articles, with 100% concordance after joint discussion of discrepancies.

Key data from each eligible article were abstracted by two independent reviewers. Data abstracted included the total number of inpatients tested and total positive for influenza by age group and year, case definition and diagnostic test, WHO region, World Bank income level (low, lower-middle, upper-middle, or high income) [11], and UN country development status (industrialized or developing) [12].

### Quality Assessment

Data quality for each eligible article was scored using a modified Newcastle–Ottawa checklist for bias assessment [13], with a score of zero or one for each of the following sources of bias: sampling process (explicit description of the sampling process for enrollment), case definition (specificity of enrollment criteria), and outcome (clarity of reported results). We explored the association between quality score and the percent positive using rank-sum non-parametric tests among all eligible articles included in the pooled analysis.

### Surveillance Data

To supplement data from published studies, we compiled data from surveillance platforms that conducted hospital-based influenza surveillance. We established a working group, the Global Respiratory Hospitalizations–Influenza Proportion Positive (GRIPP) Working Group. To be eligible, surveillance platforms needed to conduct systematic year-round inpatient enrollment, with testing for at least 12 mo and >50 pediatric patients. Forty-eight partners were contacted, of which 37 had eligible data and agreed to participate. Data were collected using a standard format. Variables included the number of persons tested and positive for influenza by calendar year and age group, as well as surveillance system information such as the total number of inpatient sites and case definition used. If surveillance data were also represented in a report identified through the systematic review, the more detailed working group dataset was used, and the published article was excluded as a duplicate source.

### Statistical Analysis

We first described the median proportion positive by age group, study duration, calendar year, number tested, diagnostic method, case definition, study population, WHO region, and country income level among all eligible datasets. We found substantial variation by age, diagnostic test, and calendar year. To reduce the influence of data from less reliable diagnostic methods (such as immunofluorescence [low sensitivity] and single serological samples [higher likelihood of false-positive findings]), we restricted the data for the meta-analysis to sites that utilized PCR for diagnosis, which is the accepted diagnostic gold standard. Further, to ensure that pooled estimates reflected seasonal rather than pandemic years, we excluded data from 2009.

Pooled estimates of the proportion of respiratory hospitalizations due to influenza were calculated by mixed regression model for each of the following age groups: <6 mo, <1 y, <2 y, <5 y, 5–17 y, and <18 y. If a dataset used age ranges that did not line up with our definitions, it was included in the smallest range that contained both bounds (e.g., a dataset from children 0–36 mo was included in the <5 y analysis). The same datasets could provide estimates for multiple age groups if they provided number tested and positive for influenza for each age group.

The mixed regression model in SAS version 9.3 (SAS Institute) used a log-linked binomial distribution of input values, and included the number tested and positive for influenza by dataset and calendar year for each age group. If a single dataset provided data by year, then each year was treated as a single observation in the model, and the dataset was defined as a cluster. Random effects were accounted for at the dataset level, irrespective of the number of observations within the dataset.

We then applied our pooled proportion positive for influenza viruses to global estimates of the total annual number of hospitalizations for ALRI among children <5 y and <1 y from Nair et al. [10] and adjusted for 2012 population to calculate the total number of influenza-associated hospitalizations in these two age groups (for which denominators were available). For children <5 y, we also applied stratified pooled estimates (by WHO region and UN country development status) to the appropriate ALRI hospitalization denominators. We then divided these total numbers of influenza-associated hospitalizations by total age-specific population (at global and regional levels, and separately for developing and industrialized countries) to estimate annual per capita influenza-associated hospitalization episodes [14].

No estimates of the frequency of ALRI among children under 6 mo existed to which we could apply the proportion positive for influenza viruses. Therefore, we calculated an incidence rate ratio of influenza-associated ALRI between children aged 0–5 mo and 6–11 mo, collected as part of a separate study [6] (S2 Appendix). We applied this ratio to our estimate of the number of influenza-associated ALRI hospitalizations in children under 1 y, assuming uniform distribution of the population under 1 y, to estimate the number of influenza-associated hospitalizations among children aged 0–5 mo.

## Results

**Fig 1:**
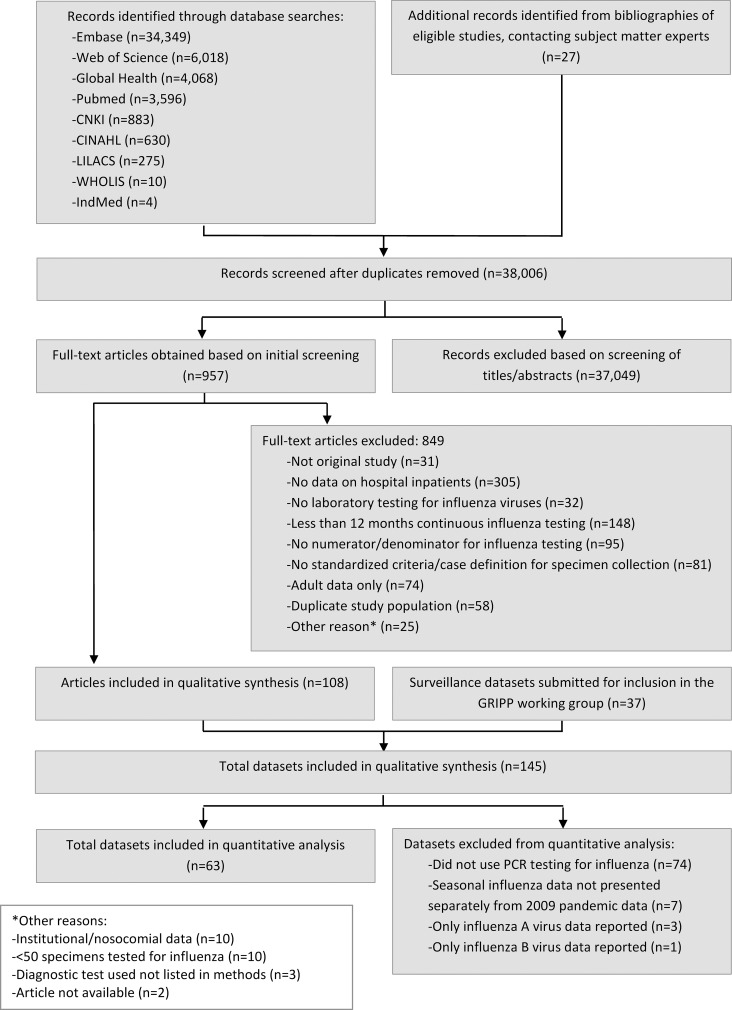
Flow diagram for systematic review process.

The systematic literature search identified 38,006 unique records from the nine scientific literature databases, of which 957 full-text articles were reviewed, and 108 included in the descriptive analysis (Fig 1) (full list of included articles provided in S1 Appendix). In addition, 37 surveillance datasets for periods ranging from 1 to 7 y, each with data from 1–49 inpatient facilities, were provided by the GRIPP Working Group. Combined, the literature search and working group resulted in a total of 145 unique data sources from 350 sites in 60 countries across all WHO regions, including southern and northern hemisphere temperate climates as well as tropical and arid regions of Asia and Africa. The two data sources combined covered a 31-y period from 1982 to 2012. More than half (55/108, 51%) of the published studies had a 3/3 quality score. Compared to published articles, surveillance datasets were more recently collected (median start year 2009 versus 2002), of longer duration (median duration 3 y versus 2 y), more likely to be PCR-based (41% versus 28%), and more likely to use the SARI case definition (84% versus 0%). Additionally, surveillance datasets were also more commonly from low-income countries compared to published articles (41% versus 6%, respectively; Table 1).

**Table 1:** Characteristics of published studies and surveillance data sources about influenza-associated respiratory illness among hospitalized children, 1982–2012.

Characteristic	Number (Percent) of Published Studies, *n =* 108, 1982–2010	Number (Percent) of Surveillance Datasets, *n =* 37, 2003–2012
**Age group** *		
<6 mo	13 (12)	12 (32)
<1 y	20 (19)	23 (62)
<2 y	19 (18)	14 (38)
<5 y	45 (42)	35 (95)
5–17 y	9 (8)	28 (76)
<18 y	63 (58)	33 (89)
**Study duration in years**		
1–2	78 (72)	16 (43)
3–4	19 (18)	13 (35)
5+	11 (10)	8 (22)
**Timeframe for outcome data** **		
Before 2009	93 (86)	12 (32)
During 2009 (“pandemic”)	1 (1)	18 (49)
After 2009	1 (1)	33 (89)
**Total cases tested**		
50–99	10 (9)	1 (3)
100–499	46 (43)	9 (24)
500–999	21 (19)	5 (14)
1,000+	31 (29)	22 (59)
**Diagnostic test**		
PCR only	30 (28)	15 (41)
Immunofluorescence only	32 (30)	0 (0)
Culture only	3 (3)	0 (0)
Multiple diagnostic tests, including PCR	14 (13)	22 (59)
Multiple diagnostic tests, excluding PCR	25 (23)	0 (0)
Other^†^	4 (4)	0 (0)
**Case definition** ^‡^		
Acute respiratory infection	35 (32)	1 (3)
ALRI	36 (33)	2 (5)
Pneumonia	18 (17)	1 (3)
SARI	0 (0)	31 (84)
Bronchiolitis	5 (5)	0 (0)
Other^§^	14 (13)	2 (5)
**Special population** ^ǁ^		
Yes	7 (6)	0 (0)
No	101 (94)	37 (100)
**WHO region**		
Africa	10 (9)	13 (35)
Americas	18 (17)	6 (16)
Eastern Mediterranean	2 (2)	2 (5)
Europe	30 (28)	2 (5)
Southeast Asia	11 (10)	6 (16)
Western Pacific	37 (34)	8 (22)
**World Bank income level**		
Low	7 (6)	15 (41)
Lower-middle	36 (33)	15 (41)
Upper-middle	20 (19)	6 (16)
High	47 (44)	1 (3)
**Quality score (published studies only)**		
0	5 (5)	
1	20 (19)	
2	28 (26)	
3	55 (51)	

*If a dataset used age ranges that did not line up with our definitions, it was included in the smallest range that contained both bounds (e.g., a dataset from children 0–36 mo was included in the <5 y analysis).

**Pandemic defined as calendar year 2009; 13 published articles provided only combined pre-pandemic/pandemic estimates.

^†^Includes serological ELISA of single or paired sera (*n =* 1) and other serological testing (*n =* 2).

^‡^Case definitions as defined per individual study criteria.

^§^Includes acute respiratory infection and/or fever (*n =* 3), acute wheezing (*n =* 2), and other criteria (*n =* 8).

^ǁ^Defined as study being designed specifically to target a high-risk population; includes recurrent wheezing/asthma (*n =* 3), intensive care unit (*n =* 2), cancer (*n =* 1), and HIV infection (*n =* 1).

The crude median percent of respiratory samples that were influenza positive among patients aged 5–17 y was more than double that among those <5 y (15%, interquartile range [IQR] 10%–22%, versus 6%, IQR 3%–9%, *p <* 0.001) and was also significantly higher in surveillance data than in published articles (9%, IQR 6%–12%, versus 5%, IQR 3%–9%, *p <* 0.01) (Table 2). The median percent positive was also significantly higher in 2009, when pandemic influenza A(H1N1)pdm09 virus emerged, and in following years, compared to before 2009 (13% in 2009, IQR 6%–20%, versus 9% after 2009, IQR 5%–11%, and 5% before 2009, IQR 3%–9%, *p <* 0.001). The majority of the datasets (57%) comprised results from PCR diagnosis. Influenza positivity was significantly lower among the datasets that used immunofluorescence alone, as compared to the datasets that used other methods such as PCR, culture, or a combination of diagnostic tests (3% versus 7%, *p <* 0.01). The frequency of influenza detection by any assay was not significantly associated with case definition used, income level, or WHO region. To account for the possibility that differences in influenza positivity by age group were due to confounding factors such as differences in PCR use or case definition applied, we analyzed these factors by age group and found no association. The studies that did not use PCR were similar to those that did use PCR by age group and timeframe of data reported. However, studies from the Americas and the Western Pacific (particularly South America and China) were less likely to use PCR, with immunofluorescence as a more common diagnostic methodology. Among published articles, there was also no difference in influenza positivity by quality score.

**Table 2:** Crude proportion of respiratory samples from hospitalized children testing positive for influenza by age group, study design, and population.

Characteristic	Number of Studies (*n =* 141)	Median Number (IQR)		Median Percent Positive (IQR)	*p*-Value*
		**Tested**	**Positive**		
**Age group** **					<0.001
<6 mo	25	386 (148–1,129)	17 (3–32)	4 (1–5)	
<1 y	42	536 (282–1,812)	34 (11–58)	4 (2–7)	
<2 y	31	506 (145–1,322)	21 (4–77)	5 (2–8)	
<5 y	80	766 (321–1,444)	42 (18–86)	6 (3–9)	
5–17 y	36	243 (90–507)	27 (14–87)	15 (10–22)	
<18 y	92	817 (239–1,524)	53 (15–110)	7 (5–12)	
**Data source**					0.001
Surveillance	37	1,159 (469–2,185)	98 (38–249)	9 (6–12)	
Published	104	435 (162–1,063)	29 (8–66)	5 (3–9)	
**Timeframe for outcome data** ^†^					<0.001
Before 2009	102	454 (170–1,086)	29 (8–70)	5 (3–9)	
During 2009 (“pandemic”)	19	610 (100–1,353)	56 (8–205)	13 (6–20)	
After 2009	34	707 (291–1,289)	64 (20–118)	9 (5–11)	
**Diagnostic test**					0.001
PCR only	44	701 (270–983)	40 (25–67)	7 (5–10)	
Immunofluorescence only	32	486 (185–1,654)	17 (8–78)	3 (2–6)	
Culture only	3	302 (68–838)	39 (5–204)	13 (7–24)	
Multiple diagnostic tests, including PCR	36	497 (185–1,167)	39 (18–88)	9 (8–10)	
Multiple diagnostic tests, excluding PCR	22	1,047 (224–2,073)	64 (15–170)	8 (5–12)	
Other	4	211 (143–1,389)	31 (7–75)	6 (4–13)	
**Case definition**					0.14
Acute respiratory infection	33	958 (415–1,429)	51 (18–88)	5 (3–10)	
ALRI	37	516 (186–1,234)	32 (17–77)	6 (3–9)	
Pneumonia	19	136 (99–627)	12 (5–32)	7 (5–9)	
SARI	31	1,159 (469–1,960)	91 (38–249)	8 (5–12)	
Bronchiolitis	5	142 (118–170)	8 (3–8)	6 (3–16)	
Other	16	278 (158–851)	29 (8–60)	7 (5–12)	
**Special population** ^‡^					0.98
No	134	701 (196–1,369)	39 (14–84)	6 (4–10)	
Yes	7	132 (119–293)	9 (7–20)	8 (2–9)	
**World Bank income level**					0.08
Low	22	806 (387–1,256)	51 (34–108)	7 (4–9)	
Lower-middle	49	808 (263–1,960)	52 (29–117)	8 (4–13)	
Upper-middle	25	455 (184–1,429)	18 (9–70)	5 (2–9)	
High	45	415 (143–1,031)	18 (7–66)	5 (5–7)	
**WHO region**					0.55
Africa	23	817 (202–1,159)	45 (16–91)	7 (4–9)	
Americas	24	299 (132–1,521)	17 (8–51)	5 (3–9)	
Eastern Mediterranean	4	1,223 (534–1,621)	40 (21–141)	8 (3–14)	
Europe	31	415 (142–916)	14 (7–66)	5 (3–7)	
Southeast Asia	17	263 (186–627)	29 (7–62)	9 (5–14)	
Western Pacific	42	1,051 (412–2,077)	67 (39–98)	7 (4–11)	

Four eligible articles provided data for influenza A only and were excluded from the overall positive analyses.

*Non-parametric comparisons conducted by Kruskal–Wallis/Wilcoxon rank-sum test. Age-based comparison conducted only between the <5 y and 5–17 y age groups.

**Age groups include datasets that include a subset within the given range, but are non-duplicative, e.g., “<5 y” includes datasets of children 0–36 mo as well as datasets of children 0–59 mo, but does not include datasets of children <1 y or <2 y.

^†^Pandemic defined as calendar year 2009.

^‡^Defined as study being designed specifically to target a high-risk population; includes recurrent wheezing/asthma (*n =* 3), intensive care unit (*n =* 2), cancer (*n =* 1), HIV infection (*n =* 1).

**Fig 2:**
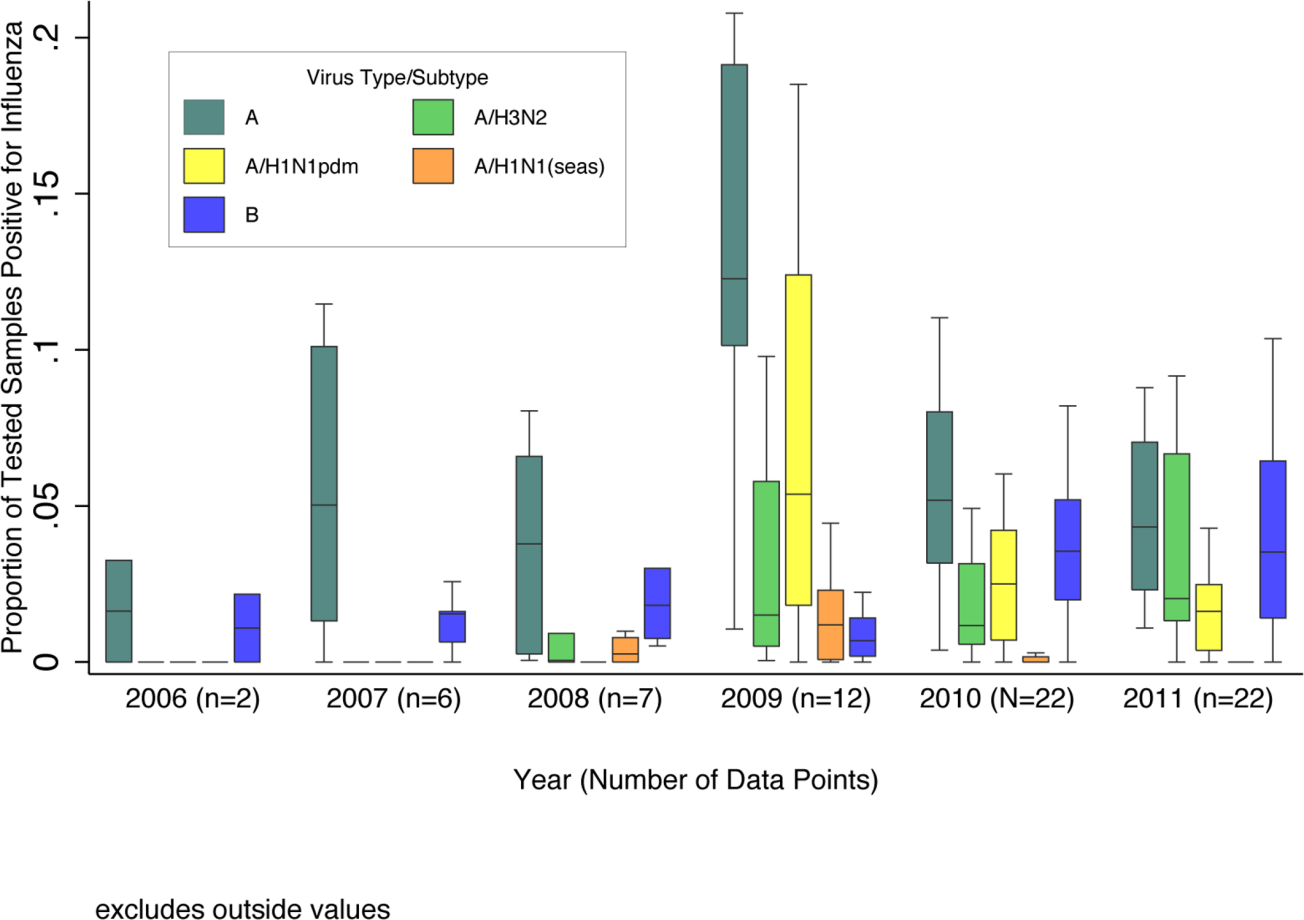
Boxplot of the proportion of pediatric (0–17 y of age) respiratory samples testing positive for influenza virus among GRIPP datasets by year and virus type/subtype. Data are for years with more than one dataset providing testing results by virus subtype. Unsubtyped influenza A viruses are included in influenza A totals, but not shown separately. Boxplot excludes outside values.

Due to steady increases in year-round hospital-based influenza surveillance over the past decade, the number of available datasets substantially increased from 2006 (*n =* 2) to 2011 (*n =* 22). The overall median influenza percent positive increased sharply from 5% in 2008 to 13% in 2009, largely driven by pandemic H1N1, the predominant subtype that year. The median total percent positive (for any influenza type/subtype) dropped substantially in 2010 to 9% and remained at 8% in 2011, as influenza A(H1N1)pdm09 circulated at lower levels and influenza A(H3N2) and B predominated (Fig 2).

The pooled meta-analyses with only PCR-confirmed data included 63 datasets, each with 1 to 6 y of data, from 41 countries. The pooled percent positive for influenza among children hospitalized with respiratory illness varied from 4.8% (95% CI 3.3%–6.9%) among those <6 mo to 16.4% (95% CI 13.6%–19.8%) among those 5–17 y, and was 9.5% (95% CI 8.1%–11.0%) overall among children <18 y. Among children <5 y, the pooled estimate was 7.4% (95% CI 6.2%–8.8%). For this age group, we also stratified the pooled estimate by region and country development status. We found the highest percent positive in Southeast Asia (8.5%, 95% CI 6.7%–10.8%) and the lowest in the Americas (4.6%, 95% CI 2.8%–7.4%), and the percent positive was lower in industrialized countries (5.9%, 95% CI 4.6%–7.5%) than in developing countries (7.7%, 95% CI 6.4%–9.3%), although these differences were not statistically significant. There were no significant differences by country income status. Post hoc sensitivity analysis of the effect of outliers (>95th percentile values) on the global estimates showed no difference.

Finally, by applying the age-specific pooled proportion to a denominator of global hospitalized ALRI episodes, we estimated the absolute number of influenza-associated hospitalizations among children <5 y to be 870,000 (95% CI 610,000 to 1,237,000), for a per capita rate of 135/100,000 children/year (95% CI 95–193) (Table 3). The influenza-associated hospitalization rate in developing countries was 3-fold that in industrialized countries, and ranged from 174/100,000 children/year in Africa to 71/100,000 children/year in the Americas. Among children <1 y, we estimated 374,000 (95% CI 264,000 to 539,000) influenza-associated hospitalizations per year worldwide.

**Table 3:** Pooled estimates of global pediatric influenza-associated hospitalizations per year, by age group, development status, and WHO region, among studies using PCR diagnostic testing.

Characteristic	*N* (Number of Countries)	Pooled Percent Positive (95% CI)	Hospitalized ALRI Episodes (Thousands)*	Global Influenza-Associated Hospitalizations (Thousands)**	Influenza-Associated Hospitalizations per 100,000 Children
**Age group**					
<6 mo	15 (14)	4.8 (3.3–6.9)	—	—	—
<1 y	26 (21)	6.1 (5.1–7.4)	6,136 (5,168–7,287)	374 (264–539)	284 (200–409)
<2 y	23 (18)	7.1 (6.1–8.4)	—	—	—
<5 y	48 (35)	7.4 (6.2–8.8)	11,751 (9,837–12,054)	870 (610–1,237)	135 (95–193)
5–17 y	27 (22)	16.4 (13.6–19.8)	—	—	—
<18 y	42 (32)	9.5 (8.1–11.0)	—	—	—
**Age <5 y by development status**					
Industrialized	7 (4)	5.9 (4.6–7.5)	551 (408–745)	33 (19–56)	48 (28–81)
Developing	41 (31)	7.7 (6.4–9.3)	11,200 (9,429–13,309)	862 (603–1,238)	150 (105–216)
**Age <5 y by WHO region**					
Africa	16 (13)	8.2 (6.4–10.6)	3,084 (1,985–4,791)	253 (127–508)	174 (87–349)
Americas	7 (5)	4.6 (2.8–7.4)	1,333 (920–1,934)	61 (26–143)	79 (33–185)
Eastern Mediterranean	1 (1)	7.4 (NA)	889 (628–1,258)	66 (46–93)^†^	95 (67–135)^†^
Europe	5 (4)	7.1 (1.5–32.7)	402 (252–642)	29 (4–210)	53 (7–387)
Southeast Asia	7 (4)	8.5 (6.7–10.8)	3,274 (2,008–5,341)	278 (135–577)	157 (76–326)
Western Pacific	12 (8)	8.5 (6.8–10.6)	2,143 (1,660–2,764)	182 (113–293)	153 (95–246)

*Determined as described by Nair et al. [10] and adjusted for 2012 population.

**Point estimates and confidence intervals were calculated as products of point estimates, lower bounds, and upper bounds (respectively) of pooled percent influenza positive and total hospitalized ALRI episodes.

^†^Since 95% CI was not calculable on the percentage influenza positive in the Eastern Mediterranean region, variance in this region’s disease burden estimates was derived only from the 95% CI of hospitalized ALRI episodes.

NA, not applicable.

The incidence of influenza-associated ALRI among children 0–5 mo was approximately 1.6 times higher than that of children 6–11 mo. Applying this rate ratio to the total influenza-associated hospitalizations for children <1 y, we estimate 228,000 (95% CI 150,000 to 344,000) influenza-associated hospitalizations per year among children aged less than 6 mo.

## Discussion

We used influenza surveillance data from 350 sites in 60 countries to estimate that 10% of global respiratory hospitalizations in children under 18 y worldwide were associated with influenza. This proportion increased by age, with the highest percentage found among school-aged children (5–17 y). We further estimated that influenza causes approximately 374,000 respiratory hospitalizations per year in children <1 y of age (including 270,000 among those less than 6 mo) and 870,000 respiratory hospitalizations per year in children <5 y of age, with the greatest impact in developing countries.

Although we did not identify any significant trends in the overall proportion positive by WHO region in the crude analysis, the final pooled estimates among children <5 y from the Americas differed from the global estimates (4.6%, 95% CI 2.8%–7.4%, versus 7.4%, 95% CI 6.2%–8.8%). This lower percentage positive for influenza may be related to the use of influenza vaccine in the region, which has been on the rise since 2004 [15]. However, vaccine coverage data among children in the region are limited, and these trends may also be a chance finding due to the small number of study sites in this region reporting results of year-round influenza testing.

Our findings on influenza burden among children <5 y are consistent with a previously published estimate using fewer datasets and a different methodological approach [6] (870,000 annual influenza-associated hospitalizations worldwide in our analysis versus 911,000 estimated by Nair et al., with overlapping confidence intervals) and demonstrate the value of using non-population-based sentinel surveillance data for disease burden estimation. Our analysis also found a substantially higher proportion of influenza-associated hospitalizations among school-aged children (5–17 y), who are an important group for transmission of influenza virus [7,16,17] and who were not included in previous global analyses. While the prevalence of influenza as a proportion of respiratory hospitalizations is lower among younger children (<5 y and particularly <1 y) than among older children, this is largely due to a high frequency of other serious respiratory pathogens such as respiratory syncytial virus (RSV) [18]. These young children experience the highest rate of total respiratory and influenza-associated hospitalizations [2,19]. Global pediatric mortality estimates suggest that the proportion of deaths caused by influenza is highest among those aged 1–12 mo (2.8% of all deaths in this age group, worldwide) [20]. Further, severe influenza infection with non-respiratory clinical presentation is not captured in our analysis, so our calculation of the total burden of influenza-associated hospitalization among the youngest children (<1 y) is likely an underestimate, as non-standard presentations (such as fever without classic respiratory signs) are known to occur in this age group [21].

Other pathogens, particularly *Streptococcus pneumoniae* and RSV, are also important causes of respiratory hospitalizations in those <5 y. Globally, it is estimated that RSV is associated with 25% of all ALRI in children <5 y, and, like influenza, RSV prevalence can vary substantially by season and across geographic locations [18]. *S*. *pneumoniae* and *Haemophilus influenzae* type B were associated with 18.3% and 4.1% of severe ALRI episodes globally among children <5 y in 2010, respectively [22], although their relative contributions to pediatric respiratory hospitalizations may vary depending on their inclusion in pediatric vaccine programs. However, a single ALRI episode may be caused by multiple pathogens, as well. For example, severe bacterial pneumonia can be a sequela of influenza infection [23]; pediatric hospitalizations for invasive pneumococcal disease in the US have been shown to increase during the 2-wk window following peak circulation of influenza, as well as other respiratory viruses [24].

Our finding of an increased impact among children of pandemic influenza compared to seasonal influenza (median estimate percent positive of 13% among children of all age groups in 2009 compared to 5% before 2009 and 9% after 2009) confirms previous findings of the effect of the pandemic on ALRI hospitalizations worldwide. In the United States, a 5-fold rise was reported in the rate of laboratory-confirmed influenza hospitalizations among children 5–17 y of age during the 2009–2010 influenza pandemic compared to non-pandemic influenza seasons [25]. Similar trends have been seen in India, where hospitalization rates due to pandemic influenza infection were particularly high among those 5–29 y, compared to other age groups [26]. Global mortality data from the 2009 pandemic suggest that the actual number of deaths was 15 times greater than laboratory-confirmed estimates, with 80% occurring in those younger than 65 y and 22% occurring in children 0–17 y [27].

Several important limitations should be considered when interpreting our findings. Differences in hospitalization practices, applications of case definitions, influenza testing practices (including sampling method), and factors such as time from symptom onset to specimen collection could make detection of influenza more or less likely and therefore bias the percent positive outcome. Our estimate of the burden of severe respiratory illness due to influenza is an underestimate of total burden for several reasons. First, our approach does not allow estimation of severe respiratory illness in individuals who did not present to hospital, which is particularly a problem in resource-limited settings with poor health care access, where hospitalization rates may be driven by proximity to a health care facility, limiting the generalizability of hospital-based burden estimates. Second, individuals with influenza virus may have stopped shedding by the time they presented to hospital and were tested, resulting in an underestimation of the true percent positive. Lastly, we assumed that influenza virus detected in individuals hospitalized for respiratory disease was causal for the hospitalization episode. While influenza is rarely found in well individuals [28], co-infections of influenza with bacteria and other viruses may result in a more severe illness, which is not explored in our analysis.

Additionally, year-by-year variability of influenza percent positive and external factors, such as co-infections and vaccination coverage, may affect influenza positivity. This year-by-year variability is better captured in the GRIPP data than in the published data, which was often not analyzable by both year and age group. Data from 2010 onward also include influenza A(H1N1)pdm09, which may have had a greater impact on immunologically naïve children, compared to other influenza viruses, during this period, resulting in an overestimate of annual influenza burden. This may be particularly the case during 2010, when WHO officially transitioned from pandemic to post-pandemic phase, especially in settings such as West Africa, where influenza A(H1N1)pdm09 only began circulating in 2010 [29]. However, no significant difference was seen in the overall pooled estimate when 2010 data were excluded, indicating that influenza A(H1N1)pdm09 activity in 2010 is not likely to impact our overall findings on proportion positive for seasonal influenza.

Our findings expand knowledge of the impact of severe influenza among children <1 and <5 y, and create an evidence base for both younger (<6 mo) and older (5–17 y) children, for whom, to our knowledge, no global estimates of influenza disease burden have been published to date. Countries considering possible influenza vaccination programs for children and/or pregnant women can use our estimates as inputs for vaccine impact and cost–benefit models. Our data may also stimulate further research into the development of effective influenza vaccines for young children. Several recent changes have made influenza vaccination a more realistic goal in settings that have not previously considered influenza vaccination programs, including 2012 WHO recommendations identifying pregnant women as the most important target group for influenza vaccination [30], as well as recent expansions in vaccine manufacturing and increasing investment into public health among middle-income countries [31]. As demand and supply grow, vaccines may drop in cost, and targeting of pediatric populations through childhood immunization programs such as the WHO Expanded Programme on Immunization could further reduce the cost of implementation of an influenza vaccine program. Maternal immunization is another strategy that would help reduce influenza burden in children less than 6 mo [32]. The development of improved influenza vaccines is needed, including products that require only one dose in young children or provide longer-term protection against a broader array of influenza viruses, such as live-attenuated and adjuvanted vaccines, as well as universal influenza vaccines that protect against multiple influenza strains. Further research is also needed to better define the burden of influenza in young children, especially those presenting with non-respiratory symptoms, possibly via approaches such as vaccine probe studies [33].

## Supporting Information

S1 Appendix Summary of published articles included in the analyses, with reference list. (DOCX)

S2 Appendix Summary of influenza-associated ALRI and total number of influenza-associated hospitalizations in children 0–5 mo and 6–11 mo, with reference list. (DOCX)

S1 Data Analysis dataset. (CSV)

S1 Fig Forest plot of data sources with PCR testing for pooled estimate, children <6 mo. (TIFF)

S2 Fig Forest plot of data sources with PCR testing for pooled estimate, children <1 y. (TIFF)

S3 Fig Forest plot of data sources with PCR testing for pooled estimate, children <2 y. (TIFF)

S4 Fig Forest plot of data sources with PCR testing for pooled estimate, children <5 y. (TIFF)

S5 Fig Forest plot of data sources with PCR testing for pooled estimate, children <18 y. (TIFF)

S6 Fig Forest plot of data sources with PCR testing for pooled estimate, children 5–17 y. (TIFF)

S1 PRISMA checklist (DOC)

S1 Table Literature search methodology and results, by database. (DOCX)

## Abbreviations

ALRI: acute lower respiratory infection
GRIPP: Global Respiratory Hospitalizations–Influenza Proportion Positive
IQR: interquartile range
RSV: respiratory syncytial virus
SARI: severe acute respiratory illness
