# Supplementary material for: Global Role and Burden of Influenza in Pediatric Respiratory Hospitalizations, 1982–2012: A Systematic Analysis
Source: PLoS Med. 2016 Mar 24;13(3):e1001977. doi: 10.1371/journal.pmed.1001977 (PMC4807087; doi:10.1371/journal.pmed.1001977)
Supplement: S1 Appendix — (DOCX) [file pmed.1001977.s001.docx]

**S1 Appendix. Summary of published articles included in the analyses, with reference list**

| **Location** | **Years** | **Case Definition** | **Age Range** | **% Influenza Positive <6 m** | **% Influenza Positive <1y** | **% Influenza Positive <2y** | **% Influenza Positive <5y** | **% Influenza Positive 5-17y** | **% Influenza Positive <18y** |
| --- | --- | --- | --- | --- | --- | --- | --- | --- | --- |
| Multiple sites, Argentina[^1^](#_ENREF_1) | 1993-1994 | Acute Lower Respiratory Infection | 0-4 years | – | – | – | 2.4 | – | – |
| Buenos Aires, Argentina[^2^](#_ENREF_2) | 2006-2006 | Reactive Airway Disease (Children with recurrent wheeze) | 0-3 years | – | – | – | 7.6 | – | – |
| Buenos Aires, Argentina[^3^](#_ENREF_3) | 1998-2002 | Acute Lower Respiratory Infection | 0-4 years | – | – | – | 2.8 | – | – |
| Vienna, Austria[^4^](#_ENREF_4) | 2000-2004 | Acute Lower Respiratory Tract Illness | 2-62 weeks | – | – | 3.4 | – | – | – |
| Mirzapur, Bangladesh[^5^](#_ENREF_5) | 1993-1996 | Pneumonia | 0-1 years | – | – | 1.7 | – | – | – |
| Salvador, Brazil[^6^](#_ENREF_6) | 2003-2005 | Community-acquired Pneumonia | 0-4 years | – | – | – | 9.2 | – | – |
| Sao Paulo, Brazil[^7^](#_ENREF_7) | 2005-2006 | Lower Respiratory Tract Illness | 0-4 years | – | – | – | 3.3 | – | – |
| Porto Alegre, Brazil[^8^](#_ENREF_8) | 2009-2010 | Acute Viral Bronchiolitis (suspected viral cause) | 0-12 months | – | 16.9 | – | – | – | – |
| Porto Alegre, Brazil[^9^](#_ENREF_9) | 1990-1992 | Acute Respiratory Infection | 0-4 years | – | – | – | 0.3 | – | – |
| Sao Paulo, Brazil[^10^](#_ENREF_10) | 2003-2003 | Lower Respiratory Tract Infection | 0-4 years | – | – | – | 5.1 | – | – |
| Sao Paulo, Brazil[^11^](#_ENREF_11) | 1999-2001 | Lower Respiratory Tract Infection (ICU patients only) | Newborns (Not further specified) | 22.2 | – | – | – | – | – |
| Sao Paulo, Brazil[^12^](#_ENREF_12) | 1995-1996 | Lower Respiratory Tract Disease | 0-14 years | – | – | – | – | – | 1.8 |
| Toronto, Canada[^13^](#_ENREF_13) | 1991-1992 | Pneumonia, Pneumonitis, Bronchiolitis, or Lower Respiratory Tract Infection | 0-5 months | 4.7 | – | – | – | – | – |
| Baffin Island, Canada[^14^](#_ENREF_14) | 2002-2003 | Lower Respiratory Tract Infection | 0-1 years | – | – | 9.1 | – | – | – |
| Conception, Chile[^15^](#_ENREF_15) | 1995-1998 | Acute Lower Respiratory Infection | 0-4 weeks | 0.0 | – | – | – | – | – |
| Qinghai, China[^16^](#_ENREF_16) | 2003-2005 | Bronchitis, Pneumonia | 0-12 years | – | – | – | – | – | 7.7 |
| Chongqing, China[^17^](#_ENREF_17) | 2006-2008 | Severe Pneumonia (ICU patients only) | 0-3 years | – | – | – | 8.4 | – | – |
| Lanzhou, China[^18^](#_ENREF_18) | 2004-2005 | Community-acquired Pneumonia | 0-14 years | – | 8.4 | – | 8.4 | 12.8 | 9.1 |
| Harbin, China[^19^](#_ENREF_19) | 2008-2008 | Acute Lower Respiratory Tract Infection | 0-15 years | – | – | – | – | – | 19.4 |
| Beijing, China[^20^](#_ENREF_20) | 2001-2005 | Acute Respiratory Infection | Children (Mostly under 5) | – | – | – | – | – | 2.3* |
| Wenzhou, China[^21^](#_ENREF_21) | 2007-2008 | Acute Respiratory Infection | 0-13 years | 0.7 | 1.0 | – | 1.5 | – | 1.5 |
| Wenzhou, China[^22^](#_ENREF_22) | 2004-2005 | Acute Lower Respiratory Tract Infection | 0-10 years | 1.3 | – | 1.1 | 1.6 | – | 1.5 |
| Jiaxing, China[^23^](#_ENREF_23) | 2007-2008 | Acute Respiratory Illness | 0-6 years | – | – | – | – | – | 0.6 |
| Suzhou, China[^24^](#_ENREF_24) | 2005-2007 | Acute Respiratory Infection | 0-14 years | 1.8 | 2.8 | – | – | – | 3.5 |
| Suzhou, China[^25^](#_ENREF_25) | 2006-2009 | Acute Respiratory Tract Infection | 0-10 years | – | – | – | – | – | 2.8 |
| Lanzhou, China[^26^](#_ENREF_26) | 2006-2009 | Acute Lower Respiratory Tract Infection | 0-14 years | 7.9 | 7.7 | – | 8.2 | 7.5 | 8.1 |
| Zhaoqing, China[^27^](#_ENREF_27) | 2003-2004 | Acute Lower Respiratory Infection | 0-12 years | – | – | 7.5 | 8.8 | 11.5 | 9.7 |
| Shuang Yashan, China[^28^](#_ENREF_28) | 2007-2009 | Acute Respiratory Illness | 0-14 years | – | 2.3 | – | 2.5 | – | 2.2 |
| Taiwan, China[^29^](#_ENREF_29) | 2001-2002 | Community-acquired Pneumonia | 0-18 years | – | – | – | – | – | 5.2 |
| Beijing, China[^30^](#_ENREF_30) | 2007-2008 | Acute Lower Respiratory Tract Infection | 0-15 years | – | 3.2 | – | 4.6 | 13.5 | 6.8 |
| Xian, China[^31^](#_ENREF_31) | 2003-2004 | Non-bacterial Pneumonia | 0-10 years | – | – | – | – | – | 12.9 |
| Shantou, China[^32^](#_ENREF_32) | 2007-2007 | Acute Lower Respiratory Tract Infection | 0-5 years | – | – | – | – | – | 11.9 |
| Wuhan, China[^33^](#_ENREF_33) | 2008-2009 | Acute Respiratory Tract Infection | 0-14 years | – | – | – | – | – | 34.8 |
| Hangzhou, China[^34^](#_ENREF_34) | 2001-2006 | Acute Lower Respiratory Tract Infection | 0-13 years | – | 1.9 | – | 2.2 | – | 2.2 |
| Taiwan, China[^35^](#_ENREF_35) | 1997-1999 | Respiratory Tract Infection | 0-12 years | – | – | – | – | – | 3.6 |
| Beijing, China[^36^](#_ENREF_36) | 2000-2006 | Acute Respiratory Infection | 0-14 years | – | – | – | – | – | 0.4 |
| Wenzhou, China[^37^](#_ENREF_37) | 2000-2003 | Acute Lower Respiratory Infection | 0-15 years | – | 1.4 | – | 1.8 | – | 1.5 |
| Changsha, China[^38^](#_ENREF_38) | 2007-2008 | Acute Lower Respiratory Tract Infection | 0-14 years | 4.8 | 5.5 | – | 5.6 | – | 5.6 |
| Aarhus, Denmark[^39^](#_ENREF_39) | 2001-2002 | Febrile episodes (Children with cancer) | 0-17 years | – | – | – | – | – | 0.5** |
| Newcastle, England[^40^](#_ENREF_40) | 1996-1998 | Community-acquired Pneumonia | 0-16 years | – | – | – | – | – | 5.2 |
| Turku, Finland[^41^](#_ENREF_41) | 2006-2007 | Community-acquired Pneumonia | 0-15 years | – | – | – | – | – | 5.3 |
| Turku, Finland[^42^](#_ENREF_42) | 2000-2002 | Acute Expiratory wheezing (Children with asthma) | 0-16 years | – | – | – | – | – | 2.4 |
| Tampere, Finland[^43^](#_ENREF_43) | 2001-2004 | Bronchiolitis | 0-5 months | 5.6 | – | – | – | – | – |
| Turku, Finland[^44^](#_ENREF_44) | 1993-1995 | Community-acquired Pneumonia | 0-15 years | – | – | – | – | – | 2.0 |
| Paris, France[^45^](#_ENREF_45) | 2002-2004 | Acute Respiratory Illness or Acute Fever | 0-16 years | – | – | – | 3.8 | – | 4.5 |
| Montpellier, France[^46^](#_ENREF_46) | 2003-2004 | Respiratory Tract Disease | 0-4 years | – | – | – | 3.1 | – | – |
| Caen, France[^47^](#_ENREF_47) | 1993-1997 | Acute exacerbations of asthma (Children with asthma) | 0-14 years | – | – | 5.6 | – | – | 5.3 |
| Tblisi, Georgia[^48^](#_ENREF_48) | 1997-2003 | Upper or Lower Respiratory Tract Infection | 0-15 years | – | – | – | – | – | 7.3 |
| Multiple sites, Germany[^49^](#_ENREF_49) | 1999-2001 | Lower Respiratory Tract Infection | 0-2 years | – | – | – | 4.2 | – | – |
| Kiel, Germany[^50^](#_ENREF_50) | 1995-1999 | Acute Respiratory Tract Infection | 0-16 years | 3.7 | 6.3 | 6.8 | 8.1 | 9.3 | 8.4 |
| Kumasi, Ghana[^51^](#_ENREF_51) | 2008-2008 | Severe Pneumonia | 0-60 months | † | – | 1.1 | 0.8 | – | – |
| Athens, Greece[^52^](#_ENREF_52) | 1999-2000 | Bronchiolitis | 0-17 months | – | – | 2.5 | – | – | – |
| Athens, Greece[^53^](#_ENREF_53) | 1999-2000 | Community-acquired Pneumonia | 5-14 years | – | – | – | – | 6.7 | – |
| Hong Kong SAR, China[^54^](#_ENREF_54) | 2003-2006 | Febrile Acute Respiratory Infection | 0-17 years | – | – | – | – | – | 14.3 |
| Hong Kong SAR, China[^55^](#_ENREF_55) | 2005-2007 | Upper Respiratory Tract Infection and/or fever, or Acute Respiratory Infection | 0-4 years | – | – | – | 11.3 | – | – |
| New Delhi, India[^56^](#_ENREF_56) | 2007-2007 | Bronchiolitis | 0-1 years | – | – | 1.2 | – | – | – |
| New Delhi, India[^57^](#_ENREF_57) | 1995-1997 | Acute Lower Respiratory Tract Illness | 0-60 months | – | – | – | 14.5 | – | – |
| Pune, India[^58^](#_ENREF_58) | 2002-2004 | Respiratory Tract Infection | Children (No range specified) | 2.0 | 2.2 | – | 1.8 | – | 1.6 |
| Beer-Sheva, Israel[^59^](#_ENREF_59) | 2001-2002 | Lower Respiratory Tract Infection | 0-4 years | – | – | – | 15.1 | – | – |
| Milan, Italy[^60^](#_ENREF_60) | 2008-2009 | Acute Respiratory Tract Infection | 0-14 years | – | – | – | – | – | 9.6 |
| Pisa, Italy[^61^](#_ENREF_61) | 2000-2006 | Acute Respiratory Disease | Infants (Not further specified) | – | – | 3.7 | – | – | – |
| Pordenone, Italy[^62^](#_ENREF_62) | 2008-2010 | Acute Respiratory Infection | Children (87% <5 years) | – | – | – | – | – | 11.8 |
| Rome, Italy[^63^](#_ENREF_63) | 2004-2007 | Acute Respiratory Infections | 0-13 years | – | – | – | – | – | 3.4 |
| Sicily, Italy[^64^](#_ENREF_64) | 2009-2010 | Hospitalized Influenza-like Illness | 0-14 years | – | – | – | 46.4* | 61.3* | 54.4* |
| Tokyo, Japan[^65^](#_ENREF_65) | 2004-2005 | Community-acquired Pneumonia | Children (89% <6 years) | – | – | – | – | – | 15.4 |
| Hokkaido, Japan[^66^](#_ENREF_66) | 2000-2001 | Community-acquired Pneumonia or Bronchitis | 0-14 years | – | – | – | – | – | 10.0* |
| Tokyo, Japan[^67^](#_ENREF_67) | 1986-1992 | Lower Respiratory Tract Illness | Children (90% <7 years) | – | – | – | – | – | 5.8 |
| Bondo, Kenya[^68^](#_ENREF_68) | 2007-2009 | Sever Acute Respiratory Illness | 0-4 years | – | – | – | 6.8 | – | – |
| Kilifi, Kenya[^69^](#_ENREF_69) | 2010-2010 | Pneumonia | 0-4 years | – | – | – | 1.5 | – | – |
| Kilifi, Kenya[^70^](#_ENREF_70) | 2007-2007 | Severe Pneumonia | 0-12 years | – | – | – | – | – | 5.9 |
| Seoul, Korea, Rep.[^71^](#_ENREF_71) | 1996-1998 | Acute Lower Respiratory Tract Infection | 0-15 years | – | – | – | – | – | 7.8 |
| Seoul, Korea, Rep.[^72^](#_ENREF_72) | 2003-2008 | Acute Respiratory Tract Infection | 0-17 years | – | – | – | – | – | 4.8 |
| Hallym, Korea, Rep.[^73^](#_ENREF_73) | 1996-1998 | Acute Respiratory Tract Infection | 0-10 years | – | – | – | – | – | 6.1 |
| Kuala Lumpur, Malaysia[^74^](#_ENREF_74) | 1999-2000 | Acute Respiratory Infection (suspected viral cause) | 0-10 years | – | – | – | – | – | 13.1 |
| Kuala Lumpur, Malaysia[^75^](#_ENREF_75) | 1982-1998 | Lower Respiratory Tract Infection | 0-24 months | 0.6 | 1.1 | 1.4 | – | – | – |
| Maputo, Mozambique[^76^](#_ENREF_76) | 2006-2007 | Severe Pneumonia | 0-4 years | – | – | – | 4.8 | – | – |
| Kathmandu, Nepal[^77^](#_ENREF_77) | 2006-2008 | Severe Pneumonia | 2-35 months | – | – | – | 7.2 | – | – |
| Ibadan, Nigeria[^78^](#_ENREF_78) | 1985-1987 | Acute Lower Respiratory Infection | 0-4 years | – | – | – | 15.6 | – | – |
| Abha, Saudi Arabia[^79^](#_ENREF_79) | 1997-2001 | Bronchiolitis | 0-5 years | – | † | † | – | – | 15.7 |
| Riyadh, Saudi Arabia[^80^](#_ENREF_80) | 1993-1996 | Acute Respiratory Tract Infection | 0-4 years | – | – | – | 3.2 | – | – |
| Singapore[^81^](#_ENREF_81) | 1998-1999 | Lower Respiratory Tract Infection | 0-14 years | – | – | – | – | – | 4.4 |
| Singapore[^82^](#_ENREF_82) | 1994-1995 | Lower Respiratory Tract Infection | 0-11 years | – | – | – | – | – | 4.5 |
| Ljubljana, Slovenia[^83^](#_ENREF_83) | 2007-2009 | Respiratory Tract Infection | 0-17 years | – | – | – | – | – | 0.9 |
| Cape Town, South Africa[^84^](#_ENREF_84) | 1995-1996 | Acute Lower Respiratory Tract Infection | 0-1 years | – | – | 0.6 | – | – | – |
| Soweto, South Africa[^85^](#_ENREF_85) | 1997-1998 | Severe lower Respiratory Tract Illness (Half of participants HIV+) | 2-60 months | – | – | – | 8.6 | – | – |
| Cape Town, South Africa[^86^](#_ENREF_86) | 2003-2004 | Respiratory Tract Infection | 0-5 years | – | – | – | – | – | 0.9 |
| Pretoria, South Africa[^87^](#_ENREF_87) | 2006-2007 | Acute Respiratory Tract Infection | 0-4 years | – | – | – | 1.7 | – | – |
| Gipuzkoa Province, Spain[^88^](#_ENREF_88) | 2004-2007 | Acute Respiratory Infection | 0-2 years | 3.6 | 4.1 | 4.9 | 4.5 | – | – |
| Barcelona, Spain[^89^](#_ENREF_89) | 2006-2007 | Acute Wheezing | 0-4 years | – | – | – | 7.4 | – | – |
| Madrid, Spain[^90^](#_ENREF_90) | 2000-2005 | Acute Respiratory Infection | 0-1 years | – | – | 5.0 | – | – | – |
| Almeira, Spain[^91^](#_ENREF_91) | 2008-2009 | Lower Respiratory Tract Infection | 0-13 years | – | – | – | – | – | 6.5 |
| Madrid, Spain[^92^](#_ENREF_92) | 2008-2010 | Respiratory Tract Disease, including asthma and wheezing | 0-13 years | – | – | – | – | – | 7.2 |
| Stockholm, Sweden[^93^](#_ENREF_93) | 2000-2004 | Acute Encephalitis | 0-18 years | – | – | – | – | – | 5.4 |
| Geneva, Switzerland[^94^](#_ENREF_94) | 2003-2005 | Community-acquired Pneumonia | 2 months-5 years | – | – | – | – | – | 14.1 |
| Bern, Switzerland[^95^](#_ENREF_95) | 1998-2010 | Respiratory Illness | 0-17 years | – | – | – | – | – | 4.6 |
| Khon Kaen, Thailand[^96^](#_ENREF_96) | 1992-1994 | Acute Lower Respiratory Infection | 0-4 years | – | 0.0 | 0.0 | 0.0 | – | – |
| Khon Kaen, Thailand[^97^](#_ENREF_97) | 2002-2004 | Acute Bronchiolitis | 1-24 months | – | – | 17.1 | – | – | – |
| Chantaburi, Thailand[^98^](#_ENREF_98) | 2005-2007 | Acute Respiratory Tract Infection | 0-14 years | – | – | – | – | – | 24.3 |
| Bangkok, Thailand[^99^](#_ENREF_99) | 2006-2007 | Community-acquired Pneumonia | 0-15 years | – | – | – | 11.3 | † | 12.5 |
| Bangkok, Thailand[^100^](#_ENREF_100) | 2004-2005 | Lower Respiratory Tract Infection or Influenza-like Illness | 0-4 years | – | – | – | 8.6 | – | – |
| Bangkok, Thailand[^101^](#_ENREF_101) | 2007-2009 | Acute Lower Respiratory Tract Infection | 1-12 months | – | 7.3 | – | – | – | – |
| Houston, United States[^102^](#_ENREF_102) | 2001-2009 | Staphylococcus aureus Pneumonia | 0-20 years | – | – | – | – | – | 7.4 |
| Houston, United States[^103^](#_ENREF_103) | 1993-1995 | Acute Respiratory Illness | 0-17 years | – | 0.0 | – | 5.4 | 4.0 | 4.9 |
| Dallas, United States[^104^](#_ENREF_104) | 1999-2000 | Lower Respiratory Infections | 0-17 years | – | – | – | – | – | 23.1 |
| Multiple sites, United States[^105^](#_ENREF_105) | 2000-2004 | Acute Respiratory Tract Infection or Fever | 0-4 years | – | – | – | 5.7 | – | – |
| Yukon Delta, United States[^106^](#_ENREF_106) | 2005-2007 | Lower Respiratory Tract Infection | 0-2 years | – | – | – | 4.9 | – | – |
| Nha Trang, Vietnam[^107^](#_ENREF_107) | 2007-2008 | Acute Respiratory Tract Infection | 0-15 | – | 19.1 | 13.6 | 15.1 | 20.8 | 15.5 |
| Ho Chi Minh City, Vietnam[^108^](#_ENREF_108) | 2004-2008 | Acute Respiratory Illness | 0-14 years | – | 10.7 | – | – | † | 16.5 |

*Estimates for Influenza A only, not included in analysis

** Estimates for Influenza B only, not included in analysis

† Less than 50 tested, data not presented or included in analysis

**References**

1. Carballal G, Videla CM, Espinosa MA, Savy V, Uez O, Sequeira MD, et al. Multicentered study of viral acute lower respiratory infections in children from four cities of Argentina, 1993-1994. Journal of medical virology. 2001; **64**(2): 167-74.

2. Maffey AF, Venialgo CM, Barrero PR, Fuse VA, Marques Mde L, Saia M, et al. [New respiratory viruses in children 2 months to 3 years old with recurrent wheeze]. Archivos argentinos de pediatria. 2008; **106**(4): 302-9.

3. Viegas M, Barrero PR, Maffey AF, Mistchenko AS. Respiratory viruses seasonality in children under five years of age in Buenos Aires, Argentina: a five-year analysis. The Journal of infection. 2004; **49**(3): 222-8.

4. Aberle JH, Aberle SW, Pracher E, Hutter HP, Kundi M, Popow-Kraupp T. Single versus dual respiratory virus infections in hospitalized infants: impact on clinical course of disease and interferon-gamma response. Pediatr Infect Dis J. 2005; **24**(7): 605-10.

5. Hasan K, Jolly P, Marquis G, Roy E, Podder G, Alam K, et al. Viral etiology of pneumonia in a cohort of newborns till 24 months of age in Rural Mirzapur, Bangladesh. Scandinavian journal of infectious diseases. 2006; **38**(8): 690-5.

6. Nascimento-Carvalho CM, Ribeiro CT, Cardoso MRA, Barral A, Araujo-Neto CA, Oliveira JR, et al. The role of respiratory viral infections among children hospitalized for community-acquired pneumonia in a developing country. Pediatric Infectious Disease Journal. 2008; **27**(10): 939-41.

7. Pecchini R, Berezin EN, Felicio MC, Passos SD, Souza MC, Lima LR, et al. Incidence and clinical characteristics of the infection by the respiratory syncytial virus in children admitted in Santa Casa de Sao Paulo Hospital. The Brazilian journal of infectious diseases : an official publication of the Brazilian Society of Infectious Diseases. 2008; **12**(6): 476-9.

8. Sparremberger DAH, Luisi F, Azevedo AV, Ribeiro AET, Wiemann AFW, de Conto BF, et al. Epidemiological surveillance and influence of co-infection by respiratory viruses in the severity of acute bronchiolitis in infants. [Portuguese]

Caracteristicas epidemiologicas e influencia da coinfeccao por virus respiratorios na gravidade da bronquiolite aguda em lactentes. Scientia Medica. 2011; **21**(3): 101-6.

9. Straliotto SM, Siqueira MM, Muller RL, Fischer GB, Cunha ML, Nestor SM. Viral etiology of acute respiratory infections among children in Porto Alegre, RS, Brazil. Revista da Sociedade Brasileira de Medicina Tropical. 2002; **35**(4): 283-91.

10. Thomazelli LM, Vieira S, Leal AL, Sousa TS, Oliveira DB, Golono MA, et al. Surveillance of eight respiratory viruses in clinical samples of pediatric patients in southeast Brazil. Jornal de pediatria. 2007; **83**(5): 422-8.

11. Vieira RA, Diniz EM, Vaz FA. Clinical and laboratory study of newborns with lower respiratory tract infection due to respiratory viruses. The journal of maternal-fetal & neonatal medicine : the official journal of the European Association of Perinatal Medicine, the Federation of Asia and Oceania Perinatal Societies, the International Society of Perinatal Obstetricians. 2003; **13**(5): 341-50.

12. Vieira SE, Stewien KE, Queiroz DA, Durigon EL, Torok TJ, Anderson LJ, et al. Clinical patterns and seasonal trends in respiratory syncytial virus hospitalizations in Sao Paulo, Brazil. Revista do Instituto de Medicina Tropical de Sao Paulo. 2001; **43**(3): 125-31.

13. Davies HD, Matlow A, Petric M, Glazier R, Wang EE. Prospective comparative study of viral, bacterial and atypical organisms identified in pneumonia and bronchiolitis in hospitalized Canadian infants. Pediatr Infect Dis J. 1996; **15**(4): 371-5.

14. Banerji A, Greenberg D, White LF, Macdonald WA, Saxton A, Thomas E, et al. Risk factors and viruses associated with hospitalization due to lower respiratory tract infections in Canadian Inuit children : a case-control study. Pediatr Infect Dis J. 2009; **28**(8): 697-701.

15. Bancalari AM, Martínez AA, Casanueva PC, Véliz Campos F, Quinteros AS, Walker BC, et al. Viral etiology of lower respiratory tract infection in newborns. Rev Chil Pediatr. 1999; **70**(3): 201-7.

16. Cao h. viral etiology studdy among bronchitis Pneumonia children at Qiang hai; 2003-2005. China women and children preventory disease medicine journal. 2006.

17. Yu CM, Yang XQ, Xu F, Zuo ZL, Zhao XD. [Analysis of viral etiology of severe pneumonia in infants and young children in Chongqing area]. Zhonghua er ke za zhi Chinese journal of pediatrics. 2010; **48**(2): 143-7.

18. Zhang Q, Guo Z, MacDonald NE. Vaccine preventable community-acquired pneumonia in hospitalized children in Northwest China. Pediatr Infect Dis J. 2011; **30**(1): 7-10.

19. Zhang HY, Li ZM, Zhang GL, Diao TT, Cao CX, Sun HQ. Respiratory viruses in hospitalized children with acute lower respiratory tract infections in harbin, China. Japanese journal of infectious diseases. 2009; **62**(6): 458-60.

20. Zhu RN, Qian Y, Wang F, Deng J, Zhao LQ, Liao B, et al. [Surveillance for influenza A virus infections in infants and young children in Beijing, China, 2001-2005]. Zhonghua er ke za zhi Chinese journal of pediatrics. 2006; **44**(7): 518-22.

21. Chang J, Li CY, Li HJ, Luo YC, Chen XF, Yang SY. [Viral etiology of acute respiratory infection in children from Wenzhou between 2007 and 2008]. Zhongguo dang dai er ke za zhi = Chinese journal of contemporary pediatrics. 2010; **12**(1): 32-4.

22. Dong L, Zhou XC, Chen XF, Yang JH, Lin J, Zhang HL, et al. [Detection of etiologic agents and antibiotic resistance in children with acute lower respiratory tract infection in Wenzhou City]. Zhongguo dang dai er ke za zhi = Chinese journal of contemporary pediatrics. 2006; **8**(5): 369-72.

23. Hu F, Xu Y, Li P, Miao Z. Viral pathogens of respiratory tract infection: a detecting assay among 2221 infants. Chinese Journal of Nosocomiology. 2010; **20**(15): 2235-7.

24. Ji W, Wu JH, Huang L, Luo YL, Zhang XL. [An etiological study on acute respiratory infection among inpatient children in Suzhou]. Zhonghua yu fang yi xue za zhi [Chinese journal of preventive medicine]. 2009; **43**(10): 867-71.

25. Ji W, Chen ZR, Guo HB, Wang MJ, Yan YD, Zhang XL, et al. [Characteristics and the prevalence of respiratory viruses and the correlation with climatic factors of hospitalized children in Suzhou children's hospital]. Zhonghua yu fang yi xue za zhi [Chinese journal of preventive medicine]. 2011; **45**(3): 205-10.

26. Jin Y, Zhang RF, Xie ZP, Yan KL, Gao HC, Song JR, et al. Newly identified respiratory viruses associated with acute lower respiratory tract infections in children in Lanzou, China, from 2006 to 2009. Clinical microbiology and infection : the official publication of the European Society of Clinical Microbiology and Infectious Diseases. 2012; **18**(1): 74-80.

27. Li h, lvbo, linzhifang, chenping. Detection and analysis of pathogens caused Children ALRI. China clinical diagnosis medicine. 2006.

28. Lihua, SongYu, Gaoyun. Influenza virus infection status on inpatient children due to ARI at shuangyashan area. China modern drug application journal. 2010.

29. Lin PY, Lin TY, Huang YC, Tsao KC, Huang YL. Human metapneumovirus and community-acquired pneumonia in children. Chang Gung medical journal. 2005; **28**(10): 683-8.

30. Liu C, Xie Z, Gonzalez R, Ren L, Kong X, Hu Y, et al. Study of viral etiology of acute lower respiratory tract infection in children. Chinese Journal of Practical Pediatrics. 2009; **24**(4): 270-3.

31. Liu Z, Wang Y, Zhang W, Luo S, Zou A. The detection and analysis of etiology of non-bacterial pneumonia in children in Xi'an. . Journal of Xi'an Jiaotong University (Medical Sciences). 2005; **26**(3): 250-2.

32. Ou SY, Lin GY, Wu Y, Lu XD, Lin CX, Zhou RB. [Viral pathogens of acute lower respiratory tract infection in hospitalized children from East Guangdong of China]. Zhongguo dang dai er ke za zhi = Chinese journal of contemporary pediatrics. 2009; **11**(3): 203-6.

33. Peng D, Zhao D, Liu J, Wang X, Yang K, Xicheng H, et al. Multipathogen infections in hospitalized children with acute respiratory infections. Virology journal. 2009; **6**: 155.

34. Tang LF, Wang TL, Tang HF, Chen ZM. Viral pathogens of acute lower respiratory tract infection in China. Indian pediatrics. 2008; **45**(12): 971-5.

35. Tsai HP, Kuo PH, Liu CC, Wang JR. Respiratory viral infections among pediatric inpatients and outpatients in Taiwan from 1997 to 1999. J Clin Microbiol. 2001; **39**(1): 111-8.

36. Wang F, Zhu RN, Qian Y, Deng J, Zhao LQ, Liao B, et al. [Surveillance for influenza B virus infections in infants and young children in Beijing, China]. Zhonghua er ke za zhi Chinese journal of pediatrics. 2008; **46**(2): 94-7.

37. Xiang Q, Luo Y, Chen X. Investigation on the viral etiology of acute lower respiratory tract infection in Wenzhou Yuying children's hospital. Chinese Journal of Practical Pediatrics. 2005; **20**(12): 738-740.

38. Xiao NG, Zhang B, Duan ZJ, Xie ZP, Zhou QH, Zhong LL, et al. [Viral etiology of 1165 hospitalized children with acute lower respiratory tract infection]. Zhongguo dang dai er ke za zhi = Chinese journal of contemporary pediatrics. 2012; **14**(1): 28-32.

39. Christensen MS, Nielsen LP, Hasle H. Few but severe viral infections in children with cancer: a prospective RT-PCR and PCR-based 12-month study. Pediatric blood & cancer. 2005; **45**(7): 945-51.

40. Drummond P, Clark J, Wheeler J, Galloway A, Freeman R, Cant A. Community acquired pneumonia - a prospective UK study. Archives of disease in childhood. 2000; **83**(5): 408-12.

41. Honkinen M, Lahti E, Osterback R, Ruuskanen O, Waris M. Viruses and bacteria in sputum samples of children with community-acquired pneumonia. Clinical microbiology and infection : the official publication of the European Society of Clinical Microbiology and Infectious Diseases. 2012; **18**(3): 300-7.

42. Jartti T, Lehtinen P, Vuorinen T, Osterback R, van den Hoogen B, Osterhaus AD, et al. Respiratory picornaviruses and respiratory syncytial virus as causative agents of acute expiratory wheezing in children. Emerg Infect Dis. 2004; **10**(6): 1095-101.

43. Nuolivirta K, Koponen P, He Q, Halkosalo A, Korppi M, Vesikari T, et al. Bordetella pertussis infection is common in nonvaccinated infants admitted for bronchiolitis. Pediatr Infect Dis J. 2010; **29**(11): 1013-5.

44. Virkki R, Juven T, Mertsola J, Ruuskanen O. Radiographic follow-up of pneumonia in children. Pediatric pulmonology. 2005; **40**(3): 223-7.

45. El-Hajje MJ, Lambe C, Moulin F, Suremain N, Pons-Catalano C, Chalumeau M, et al. The burden of respiratory viral disease in hospitalized children in Paris. European journal of pediatrics. 2008; **167**(4): 435-6.

46. Foulongne V, Guyon G, Rodiere M, Segondy M. Human metapneumovirus infection in young children hospitalized with respiratory tract disease. Pediatr Infect Dis J. 2006; **25**(4): 354-9.

47. Freymuth F, Vabret A, Brouard J, Toutain F, Verdon R, Petitjean J, et al. Detection of viral, Chlamydia pneumoniae and Mycoplasma pneumoniae infections in exacerbations of asthma in children. Journal of clinical virology : the official publication of the Pan American Society for Clinical Virology. 1999; **13**(3): 131-9.

48. Chkhaidze I, Manjavidze N, Nemsadze K. Serodiagnosis of acute respiratory infections in children in Georgia. Indian journal of pediatrics. 2006; **73**(7): 569-72.

49. Forster J, Ihorst G, Rieger CH, Stephan V, Frank HD, Gurth H, et al. Prospective population-based study of viral lower respiratory tract infections in children under 3 years of age (the PRI.DE study). European journal of pediatrics. 2004; **163**(12): 709-16.

50. Weigl JA, Puppe W, Grondahl B, Schmitt HJ. Epidemiological investigation of nine respiratory pathogens in hospitalized children in Germany using multiplex reverse-transcriptase polymerase chain reaction. European journal of clinical microbiology & infectious diseases : official publication of the European Society of Clinical Microbiology. 2000; **19**(5): 336-43.

51. Kwofie TB, Anane YA, Nkrumah B, Annan A, Nguah SB, Owusu M. Respiratory viruses in children hospitalized for acute lower respiratory tract infection in Ghana. Virology journal. 2012; **9**: 78.

52. Papadopoulos NG, Moustaki M, Tsolia M, Bossios A, Astra E, Prezerakou A, et al. Association of rhinovirus infection with increased disease severity in acute bronchiolitis. American Journal of Respiratory and Critical Care Medicine. 2002; **165**(9): 1285-9.

53. Tsolia MN, Psarras S, Bossios A, Audi H, Paldanius M, Gourgiotis D, et al. Etiology of community-acquired pneumonia in hospitalized school-age children: Evidence for high prevalence of viral infections. Clinical Infectious Diseases. 2004; **39**(5): 681-6.

54. Chiu SS, Chan KH, Chen H, Young BW, Lim W, Wong WH, et al. Virologically confirmed population-based burden of hospitalization caused by influenza A and B among children in Hong Kong. Clin Infect Dis. 2009; **49**(7): 1016-21.

55. Tsung LY, Choi KC, Nelson EA, Chan PK, Sung RY. Factors associated with length of hospital stay in children with respiratory disease. Hong Kong medical journal = Xianggang yi xue za zhi / Hong Kong Academy of Medicine. 2010; **16**(6): 440-6.

56. Kaur C, Chohan S, Khare S, Puliyel JM. Respiratory viruses in acute bronchiolitis in Delhi. Indian pediatrics. 2010; **47**(4): 342-3.

57. Maitreyi RS, Broor S, Kabra SK, Ghosh M, Seth P, Dar L, et al. Rapid detection of respiratory viruses by centrifugation enhanced cultures from children with acute lower respiratory tract infections. Journal of clinical virology : the official publication of the Pan American Society for Clinical Virology. 2000; **16**(1): 41-7.

58. Yeolekar LR, Damle RG, Kamat AN, Khude MR, Simha V, Pandit AN. Respiratory viruses in acute respiratory tract infections in Western India. Indian journal of pediatrics. 2008; **75**(4): 341-5.

59. Wolf DG, Greenberg D, Kalkstein D, Shemer-Avni Y, Givon-Lavi N, Saleh N, et al. Comparison of human metapneumovirus, respiratory syncytial virus and influenza A virus lower respiratory tract infections in hospitalized young children. Pediatr Infect Dis J. 2006; **25**(4): 320-4.

60. Zuccotti G, Dilillo D, Zappa A, Galli E, Amendola A, Martinelli M, et al. Epidemiological and clinical features of respiratory viral infections in hospitalized children during the circulation of influenza virus A(H1N1) 2009. Influenza Other Respi Viruses. 2011; **5**(6): e528-34.

61. Maggi F, Andreoli E, Pifferi M, Meschi S, Rocchi J, Bendinelli M. Human bocavirus in Italian patients with respiratory diseases. Journal of clinical virology : the official publication of the Pan American Society for Clinical Virology. 2007; **38**(4): 321-5.

62. Modolo ML, Carnelutto V, Lucchese T, Zamparo S, Avolio M, De Rosa R, et al. Epidemiology of viral infections in acute respiratory illnesses in children. Clinical Microbiology and Infection. 2011; **17**: S732.

63. Pierangeli A, Scagnolari C, Trombetti S, Grossi R, Battaglia M, Moretti C, et al. Human bocavirus infection in hospitalized children in Italy. Influenza Other Respi Viruses. 2008; **2**(5): 175-9.

64. Tramuto F, Maida CM, Bonura F, Perna AM, Puzelli S, De Marco MA, et al. Surveillance of hospitalised patients with influenza-like illness during pandemic influenza A(H1N1) season in Sicily, April 2009-December 2010. Euro surveillance : bulletin europeen sur les maladies transmissibles = European communicable disease bulletin. 2011; **16**(35).

65. Nakayama E, Hasegawa K, Morozumi M, Kobayashi R, Chiba N, Iitsuka T, et al. Rapid optimization of antimicrobial chemotherapy given to pediatric patients with community-acquired pneumonia using PCR techniques with serology and standard culture. Journal of infection and chemotherapy : official journal of the Japan Society of Chemotherapy. 2007; **13**(5): 305-13.

66. Numazaki K, Chiba S, Umetsu M, Tanaka T, Yoshimura H, Kuniya Y, et al. Etiological agents of lower respiratory tract infections in Japanese children. In vivo. 2004; **18**(1): 67-71.

67. Sonoda S, Gotoh Y, Bann F, Nakayama T. Acute lower respiratory infections in hospitalized children over a 6 year period in Tokyo. Pediatrics international : official journal of the Japan Pediatric Society. 1999; **41**(5): 519-24.

68. Feikin DR, Ope MO, Aura B, Fuller JA, Gikunju S, Vulule J, et al. The population-based burden of influenza-associated hospitalization in rural western Kenya, 2007-2009. Bulletin of the World Health Organization. 2012; **90**(4): 256-63A.

69. Hammitt LL, Kazungu S, Morpeth SC, Gibson DG, Mvera B, Brent AJ, et al. A Preliminary Study of Pneumonia Etiology Among Hospitalized Children in Kenya. Clinical Infectious Diseases. 2012; **54**: S190-S9.

70. Berkley JA, Munywoki P, Ngama M, Kazungu S, Abwao J, Bett A, et al. Viral etiology of severe pneumonia among Kenyan infants and children. Jama. 2010; **303**(20): 2051-7.

71. Ahn KM, Chung SH, Chung EH, Koh YJ, Nam SY, Kim JH, et al. Clinical characteristics of acute viral lower respiratory tract infections in hospitalized children in Seoul, 1996-1998. Journal of Korean medical science. 1999; **14**(4): 405-11.

72. Kim CK, Choi J, Callaway Z, Kim HB, Chung JY, Koh YY, et al. Clinical and epidemiological comparison of human metapneumovirus and respiratory syncytial virus in seoul, Korea, 2003-2008. Journal of Korean medical science. 2010; **25**(3): 342-7.

73. Kim MR, Lee HR, Lee GM. Epidemiology of acute viral respiratory tract infections in Korean children. The Journal of infection. 2000; **41**(2): 152-8.

74. Zamberi S, Zulkifli I, Ilina I. Respiratory viruses detected in hospitalised paediatric patients with respiratory infections. The Medical journal of Malaysia. 2003; **58**(5): 681-7.

75. Chan PW, Goh AY, Chua KB, Kharullah NS, Hooi PS. Viral aetiology of lower respiratory tract infection in young Malaysian children. Journal of paediatrics and child health. 1999; **35**(3): 287-90.

76. O'Callaghan-Gordo C, Bassat Q, Morais L, Diez-Padrisa N, Machevo S, Nhampossa T, et al. Etiology and Epidemiology of Viral Pneumonia Among Hospitalized Children in Rural Mozambique A Malaria Endemic Area With High Prevalence of Human Immunodeficiency Virus. Pediatric Infectious Disease Journal. 2011; **30**(1): 39-44.

77. Mathisen M, Basnet S, Sharma A, Shrestha PS, Sharma BN, Valentiner-Branth P, et al. RNA viruses in young Nepalese children hospitalized with severe pneumonia. Pediatr Infect Dis J. 2011; **30**(12): 1032-6.

78. Johnson AWBR, Osinusi K, Aderele WI, Gbadero DA, Olaleye OD, Adeyemi-Doro FAB. Etiologic agents and outcome determinants of community-acquired pneumonia in urban children: A hospital-based study. Journal of the National Medical Association. 2008; **100**(4): 370-85.

79. Al-Shehri MA, Sadeq A, Quli K. Bronchiolitis in Abha, Southwest Saudi Arabia: viral etiology and predictors for hospital admission. West African journal of medicine. 2005; **24**(4): 299-304.

80. Bakir TM, Halawani M, Ramia S. Viral aetiology and epidemiology of acute respiratory infections in hospitalized Saudi children. Journal of tropical pediatrics. 1998; **44**(2): 100-3.

81. Yin CC, Huah LW, Lin JT, Goh A, Ling H, Moh CO. Lower respiratory tract infection in hospitalized children. Respirology. 2003; **8**(1): 83-9.

82. Chong CY, Lim WH, Heng JT, Chay OM. The changing trend in the pattern of infective etiologies in childhood acute lower respiratory tract infection. Acta Paediatrica Japonica. 1997; **39**(3): 317-21.

83. Ursic T, Jevsnik M, Zigon N, Krivec U, Beden AB, Praprotnik M, et al. Human bocavirus and other respiratory viral infections in a 2-year cohort of hospitalized children. Journal of medical virology. 2012; **84**(1): 99-108.

84. Hussey GD, Apolles P, Arendse Z, Yeates J, Robertson A, Swingler G, et al. Respiratory syncytial virus infection in children hospitalised with acute lower respiratory tract infection. South African medical journal = Suid-Afrikaanse tydskrif vir geneeskunde. 2000; **90**(5): 509-12.

85. Madhi SA, Schoub B, Simmank K, Blackburn N, Klugman KP. Increased burden of respiratory viral associated severe lower respiratory tract infections in children infected with human immunodeficiency virus type-1. The Journal of pediatrics. 2000; **137**(1): 78-84.

86. Smuts H. Human coronavirus NL63 infections in infants hospitalised with acute respiratory tract infections in South Africa. Influenza Other Respi Viruses. 2008; **2**(4): 135-8.

87. Venter M, Lassauniere R, Kresfelder TL, Westerberg Y, Visser A. Contribution of common and recently described respiratory viruses to annual hospitalizations in children in South Africa. Journal of medical virology. 2011; **83**(8): 1458-68.

88. Cilla G, Onate E, Perez-Yarza EG, Montes M, Vicente D, Perez-Trallero E. Hospitalization rates for human metapneumovirus infection among 0- to 3-year-olds in Gipuzkoa (Basque Country), Spain. Epidemiol Infect. 2009; **137**(1): 66-72.

89. Fuenzalida L, Fabrega J, Blanco S, Del Mar Martinez M, Prat C, Perez M, et al. Usefulness of two new methods for diagnosing metapneumovirus infections in children. Clinical microbiology and infection : the official publication of the European Society of Clinical Microbiology and Infectious Diseases. 2010; **16**(11): 1663-8.

90. Garcia-Garcia ML, Calvo C, Perez-Brena P, De Cea JM, Acosta B, Casas I. Prevalence and clinical characteristics of human metapneumovirus infections in hospitalized infants in Spain. Pediatric pulmonology. 2006; **41**(9): 863-71.

91. Sanchez-Yebra W, Avila-Carrillo JA, Gimenez-Sanchez F, Reyes-Bertos A, Sanchez-Forte M, Morales-Torres M, et al. Viral agents causing lower respiratory tract infections in hospitalized children: evaluation of the Speed-Oligo(R) RSV assay for the detection of respiratory syncytial virus. European journal of clinical microbiology & infectious diseases : official publication of the European Society of Clinical Microbiology. 2012; **31**(3): 243-50.

92. Calvo C, Garcia-Garcia ML, Ambrona P, Rico M, Pozo F, Del Mar Molinero M, et al. The burden of infections by parainfluenza virus in hospitalized children in Spain. Pediatr Infect Dis J. 2011; **30**(9): 792-4.

93. Fowler A, Stodberg T, Eriksson M, Wickstrom R. Childhood encephalitis in Sweden: Etiology, clinical presentation and outcome. European Journal of Paediatric Neurology. 2008; **12**(6): 484-90.

94. Cevey-Macherel M, Galetto-Lacour A, Gervaix A, Siegrist CA, Bille J, Bescher-Ninet B, et al. Etiology of community-acquired pneumonia in hospitalized children based on WHO clinical guidelines. European journal of pediatrics. 2009; **168**(12): 1429-36.

95. Sadeghi CD, Aebi C, Gorgievski-Hrisoho M, Muhlemann K, Barbani MT. Twelve years' detection of respiratory viruses by immunofluorescence in hospitalised children: impact of the introduction of a new respiratory picornavirus assay. BMC Infect Dis. 2011; **11**: 41.

96. Ekalaksananan T, Pientong C, Kongyingyoes B, Pairojkul S, Teeratakulpisarn J, Heng S. Etiology of acute lower respiratory tract infection in children at Srinagarind Hospital, Khon Kaen, Thailand. The Southeast Asian journal of tropical medicine and public health. 2001; **32**(3): 513-9.

97. Pientong C, Ekalaksananan T, Teeratakulpisarn J, Tanuwattanachai S, Kongyingyoes B, Limwattananon C. Atypical bacterial pathogen infection in children with acute bronchiolitis in northeast Thailand. Journal of microbiology, immunology, and infection = Wei mian yu gan ran za zhi. 2011; **44**(2): 95-100.

98. Prasarnphanich T. The accuracy of clinical diagnosis of influenza in Thai children. Journal of Pediatric Infectious Diseases. 2010; **5**(2): 155-9.

99. Samransamruajkit R, Hiranrat T, Chieochansin T, Sritippayawan S, Deerojanawong J, Prapphal N, et al. Prevalence, clinical presentations and complications among hospitalized children with influenza pneumonia. Japanese journal of infectious diseases. 2008; **61**(6): 446-9.

100. Suntarattiwong P, Sian-nork C, Thongtipa P, Thawatsupha P, Kitphati R, Chotpitayasunondh T. Influenza-associated hospitalization in urban Thai children. Influenza Other Respi Viruses. 2007; **1**(5-6): 177-82.

101. Suntarattiwong P, Sojisirikul K, Sitaposa P, Pornpatanangkoon A, Chittaganpitch M, Srijuntongsiri S, et al. Clinical and epidemiological characteristics of respiratory syncytial virus and influenza virus associated hospitalization in urban Thai infants. J Med Assoc Thai. 2011; **94 Suppl 3**: S164-71.

102. Carrillo-Marquez MA, Hulten KG, Hammerman W, Lamberth L, Mason EO, Kaplan SL. Staphylococcus aureus Pneumonia in Children in the Era of Community-acquired Methicillin-resistance at Texas Children's Hospital. Pediatric Infectious Disease Journal. 2011; **30**(7): 545-50.

103. Atmar RL, Piedra PA, Patel SM, Greenberg SB, Couch RB, Glezen WP. Picornavirus, the most common respiratory virus causing infection among patients of all ages hospitalized with acute respiratory illness. J Clin Microbiol. 2012; **50**(2): 506-8.

104. Michelow IC, Olsen K, Lozano J, Rollins NK, Duffy LB, Ziegler T, et al. Epidemiology and clinical characteristics of community-acquired pneumonia in hospitalized children. Pediatrics. 2004; **113**(4): 701-7.

105. Poehling KA, Edwards KM, Weinberg GA, Szilagyi P, Staat MA, Iwane MK, et al. The underrecognized burden of influenza in young children. N Engl J Med. 2006; **355**(1): 31-40.

106. Singleton RJ, Bulkow LR, Miernyk K, DeByle C, Pruitt L, Hummel KB, et al. Viral respiratory infections in hospitalized and community control children in Alaska. Journal of medical virology. 2010; **82**(7): 1282-90.

107. Yoshida LM, Suzuki M, Yamamoto T, Nguyen HA, Nguyen CD, Nguyen AT, et al. Viral pathogens associated with acute respiratory infections in central vietnamese children. Pediatr Infect Dis J. 2010; **29**(1): 75-7.

108. Do AH, van Doorn HR, Nghiem MN, Bryant JE, Hoang TH, Do QH, et al. Viral etiologies of acute respiratory infections among hospitalized Vietnamese children in Ho Chi Minh City, 2004-2008. PloS one. 2011; **6**(3): e18176.
