# Supplementary material for: Global Role and Burden of Influenza in Pediatric Respiratory Hospitalizations, 1982–2012: A Systematic Analysis
Source: PLoS Med. 2016 Mar 24;13(3):e1001977. doi: 10.1371/journal.pmed.1001977 (PMC4807087; doi:10.1371/journal.pmed.1001977)
Supplement: S2 Appendix — (DOCX) [file pmed.1001977.s002.docx]

**S2 Appendix. Summary of influenza-associated ALRI* and total number of influenza-associated hospitalizations in children 0-5 months and 6-11 months, with reference list**

| **Name of Study** | **Study Period** | **Influenza-associated ALRI per 100,000 person-years (95% CI)**  **<6 Months** | **Influenza-associated ALRI per 100,000 person-years**  **(95% CI)**  **6-11 Months** |
| --- | --- | --- | --- |
| Kamalapur, Bangladesh (Brooks and colleagues, unpublished) | 2008 | 264 (37-1872) | -** |
| Bondo district, Kenya (Ope and colleagues, unpublished) | 2007-2009 | 107 (59-193) | 135 (80-227) |
| Kilifi district, Kenya (Berkley and colleagues, unpublished) | 2007 | 372 (232-599) | 257 (143-465) |
| Lwak, Kisumu, Kenya (Mott and colleagues, unpublished) | 2008 | 398 (128-1234) | -** |
| Manhiça district, Mozambique (Roca and colleagues, unpublished) | 2006-2007 | 420 (189-935) | 280 (105-747) |
| Bohol, Philippines (Lucero and colleagues, unpublished) | 2000-2004 | 239 (128-444) | 237 (141-401) |
| Sa Kaeo and Nakhon Phanom, Thailand (Olsen and colleagues, unpublished) | 2005-2008 | 369 (306-446) | 816 (726-917) |
| East London, United Kingdom (Ajayi-Obe et al.,2008)[^1^](#_ENREF_1) | 2002-2004 | 442 (259-755) | 137 (53-359) |
| Multistate, USA (Dawood et al., 2010)[^2^](#_ENREF_2) | 2003-2008 | 160 (151-170) | 65 (60-72) |
| Colorado, USA; Mixed (Simoes and colleagues, unpublished) | 2000-2008 | 243 (226-261) | 142 (129-156) |
| Davidson County, Tennessee, USA (Grijalva et al., 2006)[^3^](#_ENREF_3) | 2003-04 | 910 (670-1450) | 25 (3-175) |
| Davidson County (Tennessee), Monroe County (New York) and Hamiltion County (Ohio), USA (Grijalva et al., 2007)[^4^](#_ENREF_4) | 2004-05 | 438 (389-521) | 264 (192-363) |
| Salt Lake County, Utah, USA (Ampofo et al., 2006)[^5^](#_ENREF_5) | 2001-2004 | 253 (161-375) | 331 (230-477) |
| **Median incidence of influenza-associated ALRI per 100,000 population** **(95% CI)** |  | 369 (239–438) | 237 (135–331) |
| **Global influenza-associated hospitalizations (thousands)** † |  | 228 (150–344) | 146 (95–245) |

* Collected as described by Nair, et al.[^6^](#_ENREF_6)

**No data available

† Based on incidence rate ratio applied to total hospitalizations <1 year of age (374,000, 95% CI: 264,000–539,000)

**References**

1. Ajayi-Obe EK, Coen PG, Handa R, Hawrami K, Aitken C, McIntosh ED, et al. Influenza A and respiratory syncytial virus hospital burden in young children in East London. Epidemiol Infect. 2008; **136**(8): 1046-58.

2. Dawood FS, Fiore A, Kamimoto L, Nowell M, Reingold A, Gershman K, et al. Influenza-associated pneumonia in children hospitalized with laboratory-confirmed influenza, 2003-2008. Pediatr Infect Dis J. 2010; **29**(7): 585-90.

3. Grijalva CG, Craig AS, Dupont WD, Bridges CB, Schrag SJ, Iwane MK, et al. Estimating influenza hospitalizations among children. Emerg Infect Dis. 2006; **12**(1): 103-9.

4. Grijalva CG, Weinberg GA, Bennett NM, Staat MA, Craig AS, Dupont WD, et al. Estimating the undetected burden of influenza hospitalizations in children. Epidemiol Infect. 2007; **135**(6): 951-8.

5. Ampofo K, Gesteland PH, Bender J, Mills M, Daly J, Samore M, et al. Epidemiology, complications, and cost of hospitalization in children with laboratory-confirmed influenza infection. Pediatrics. 2006; **118**(6): 2409-17.

6. Nair H, Brooks WA, Katz M, Roca A, Berkley JA, Madhi SA, et al. Global burden of respiratory infections due to seasonal influenza in young children: a systematic review and meta-analysis. Lancet. 2011; **378**(9807): 1917-30.
