# Supplementary figures and images for: Global Role and Burden of Influenza in Pediatric Respiratory Hospitalizations, 1982–2012: A Systematic Analysis
Source: PLoS Med. 2016 Mar 24;13(3):e1001977. doi: 10.1371/journal.pmed.1001977 (PMC4807087; doi:10.1371/journal.pmed.1001977)

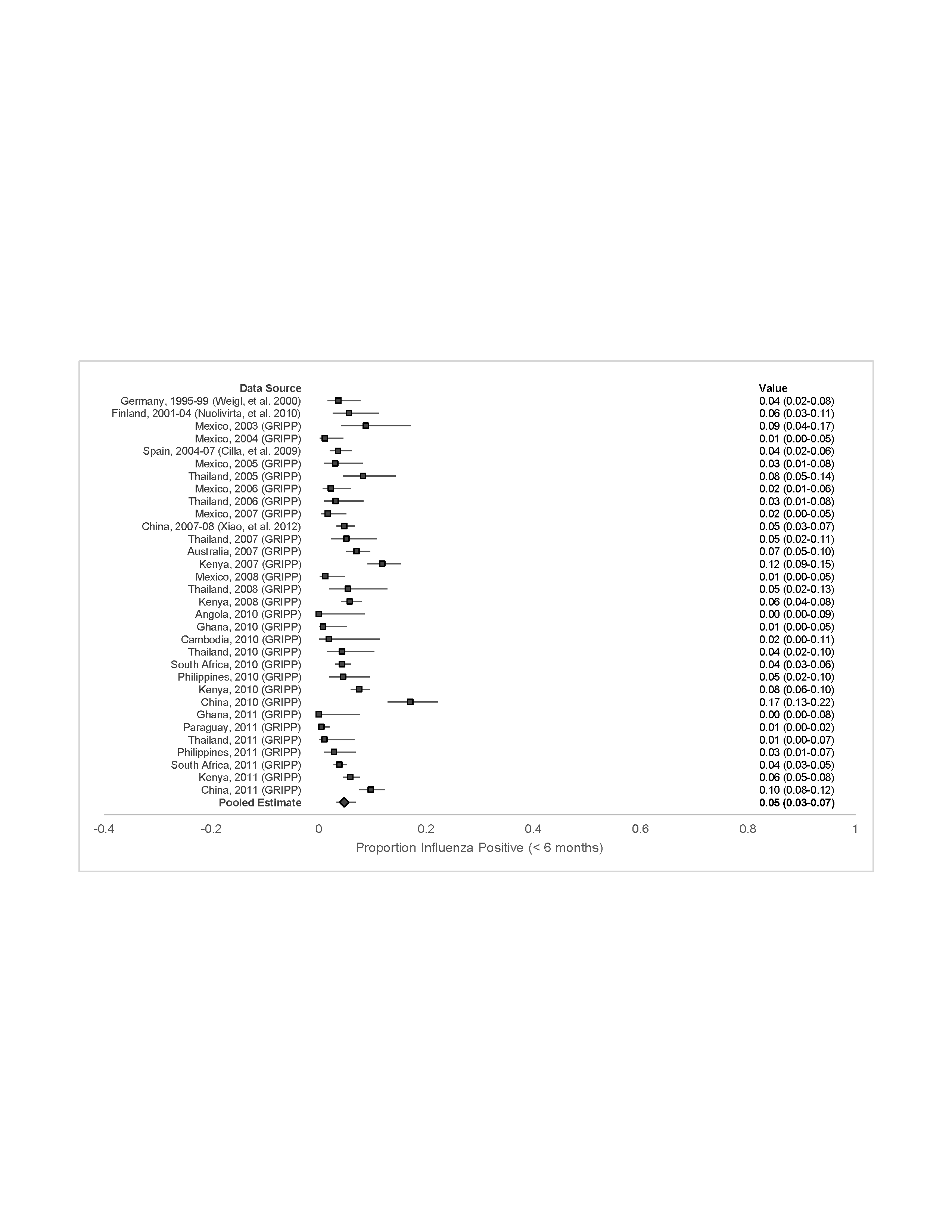

Supplement: S1 Fig — (TIFF) [file pmed.1001977.s004.tiff]

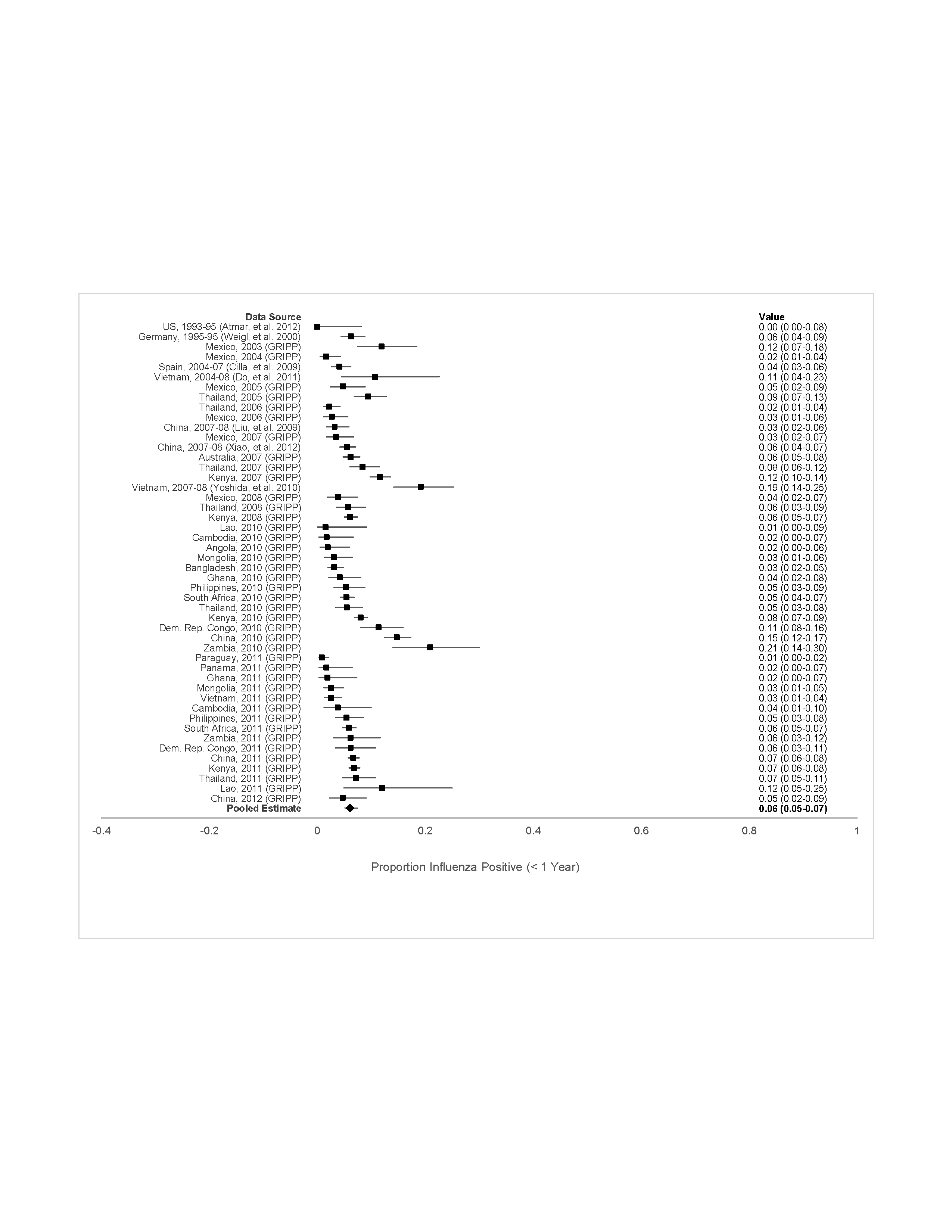

Supplement: S2 Fig — (TIFF) [file pmed.1001977.s005.tiff]

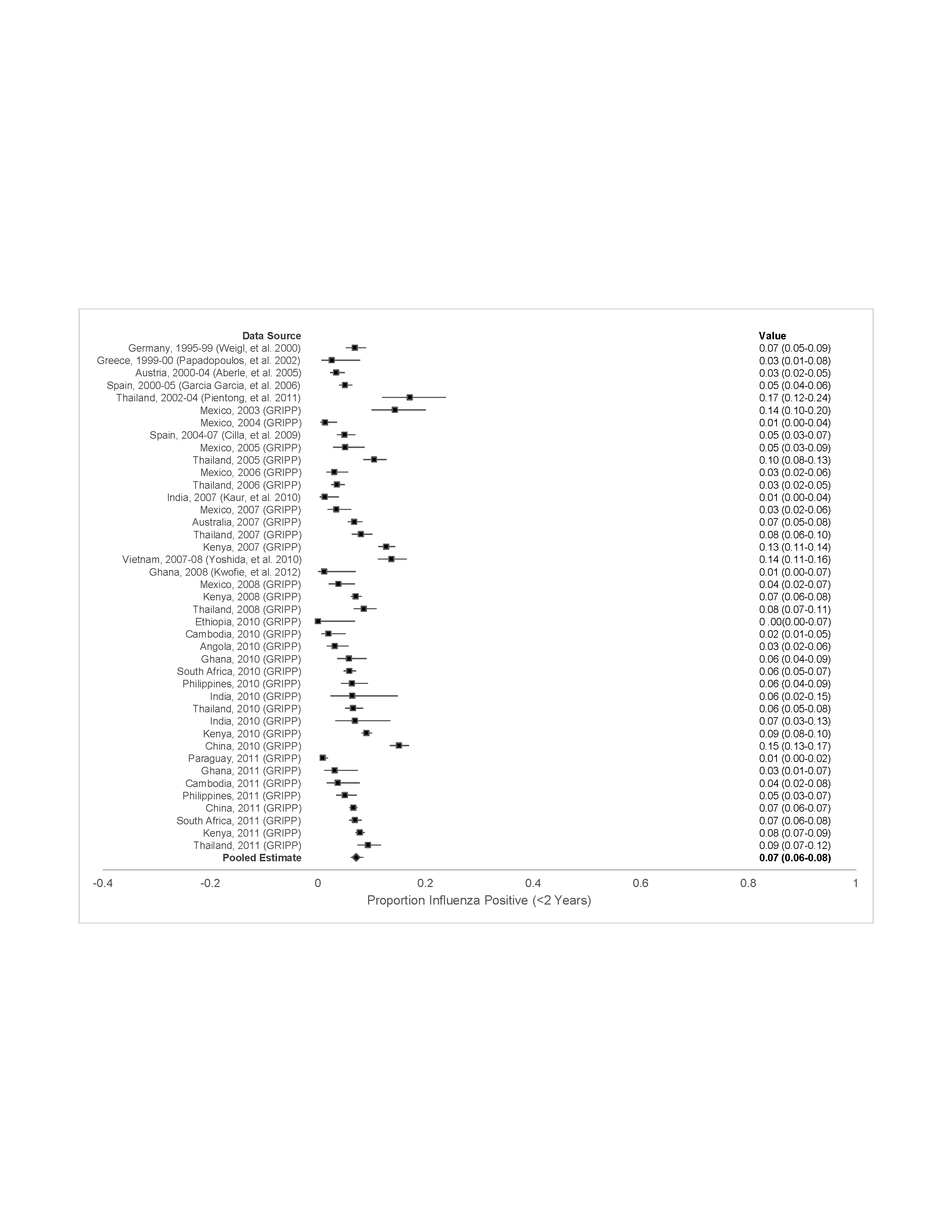

Supplement: S3 Fig — (TIFF) [file pmed.1001977.s006.tiff]

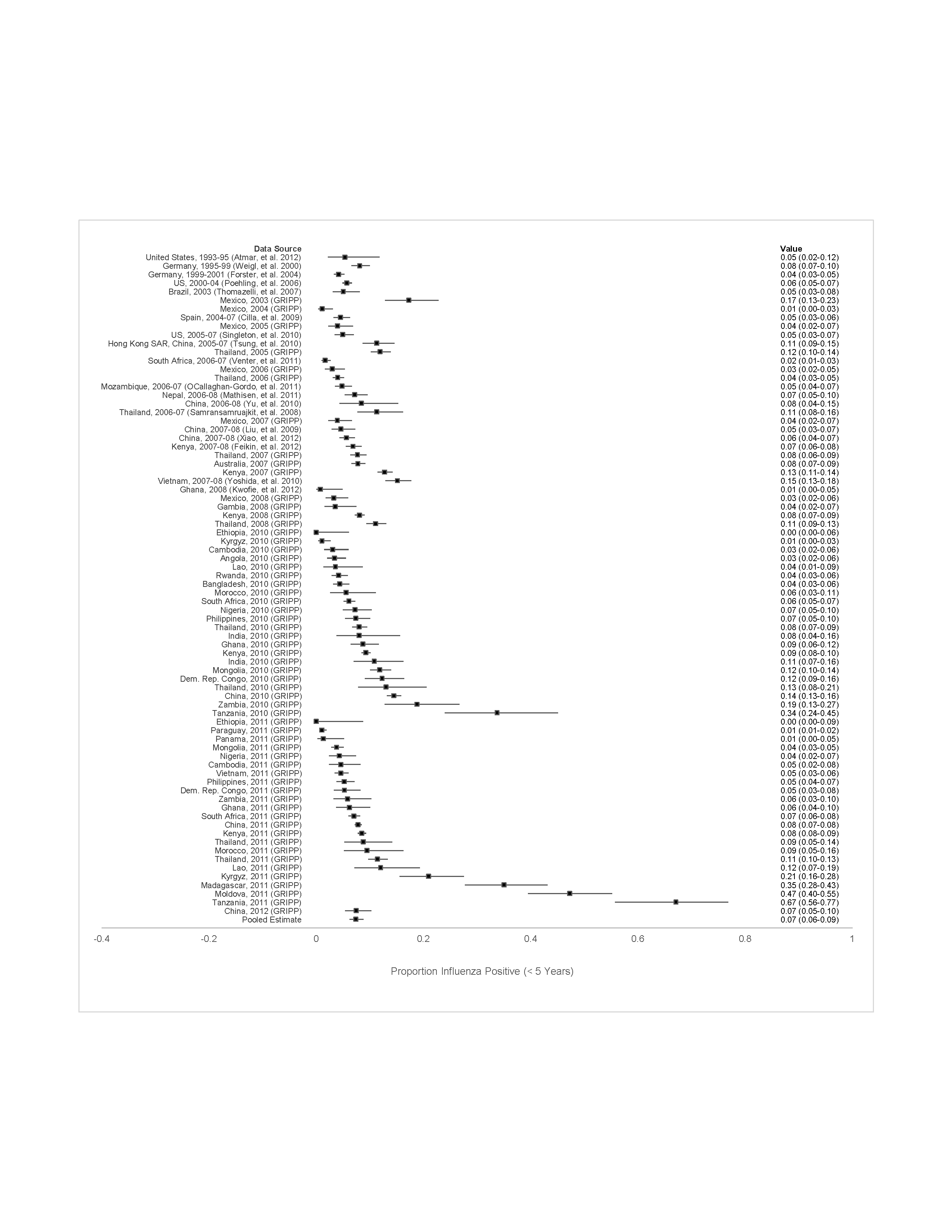

Supplement: S4 Fig — (TIFF) [file pmed.1001977.s007.tiff]

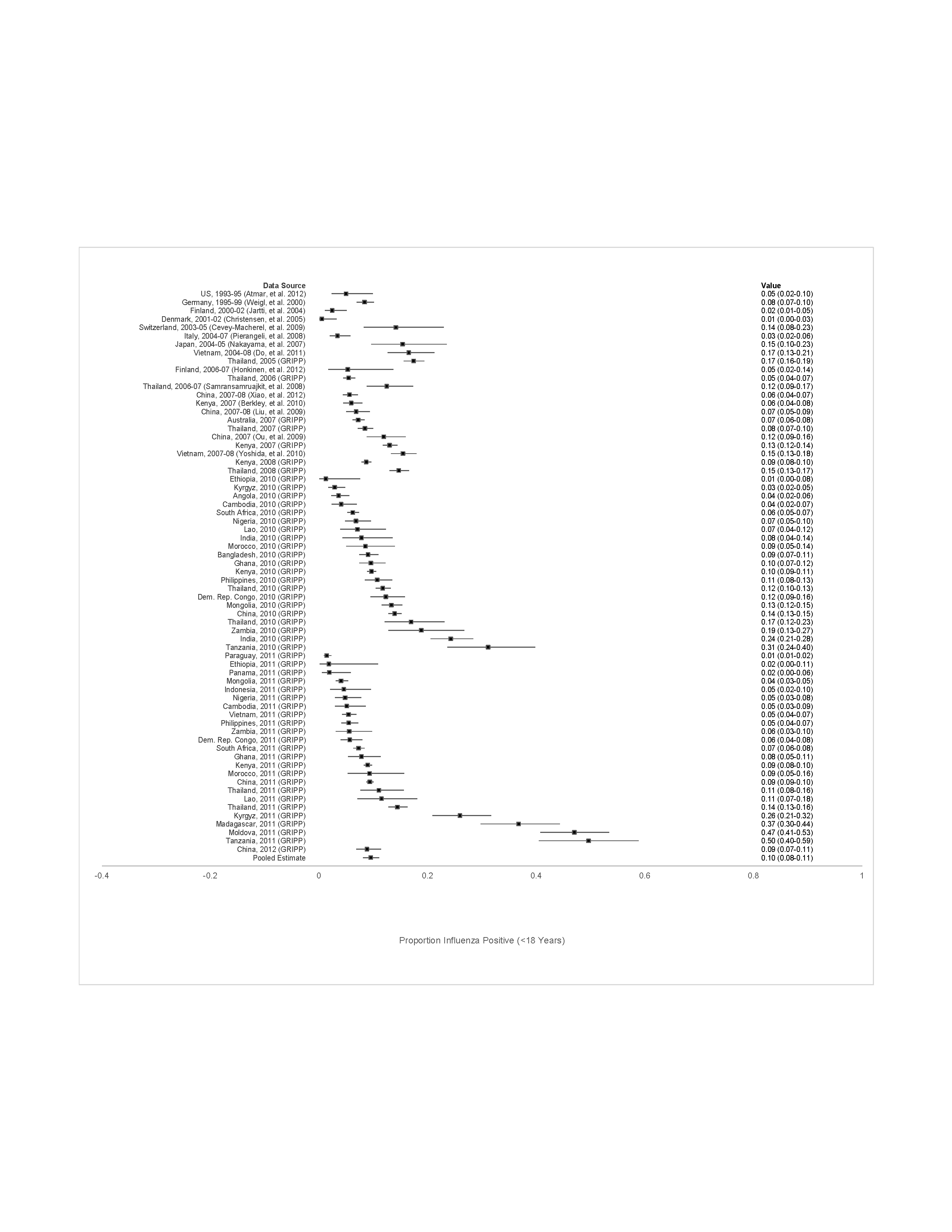

Supplement: S5 Fig — (TIFF) [file pmed.1001977.s008.tiff]

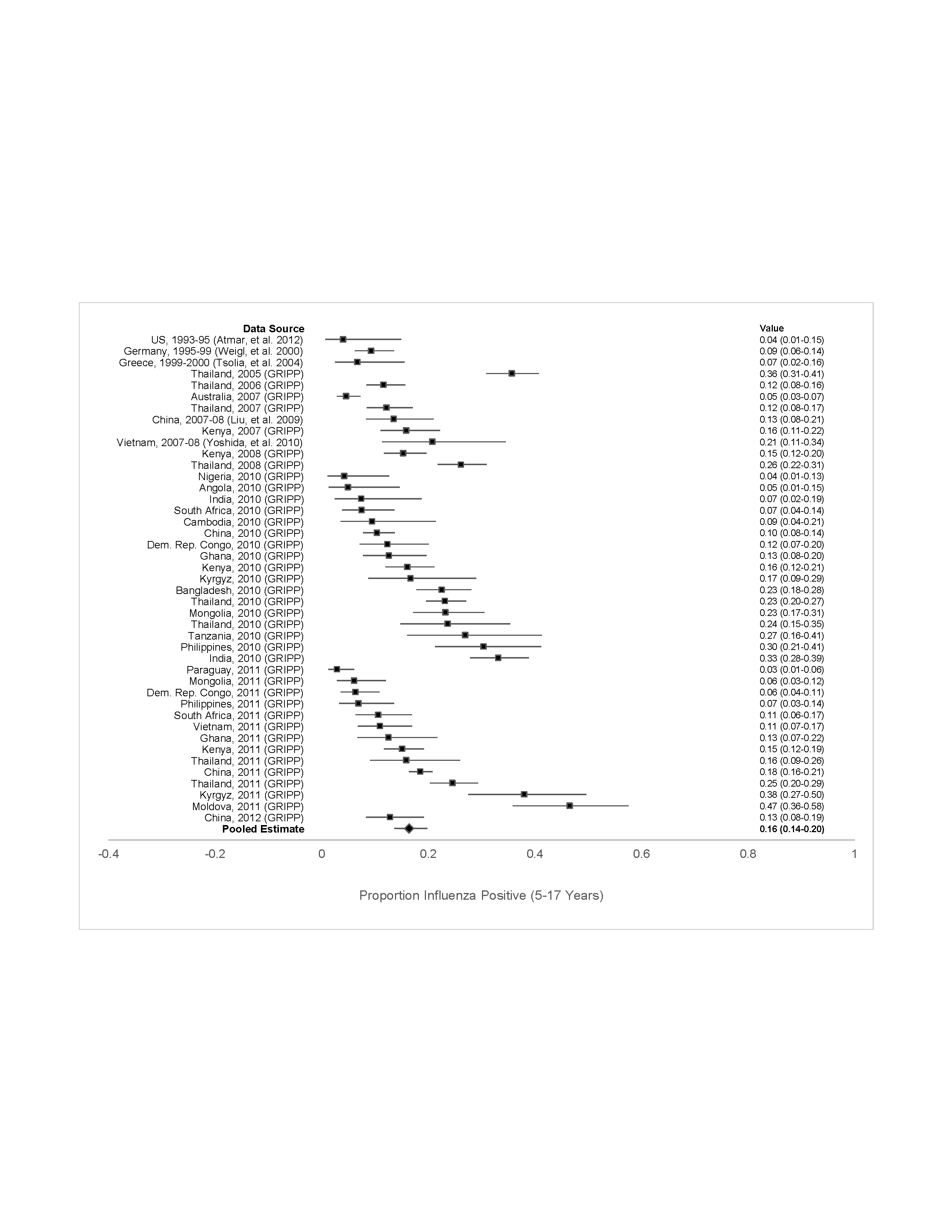

Supplement: S6 Fig — (TIFF) [file pmed.1001977.s009.tiff]
