# Supplementary material for: Global Role and Burden of Influenza in Pediatric Respiratory Hospitalizations, 1982–2012: A Systematic Analysis
Source: PLoS Med. 2016 Mar 24;13(3):e1001977. doi: 10.1371/journal.pmed.1001977 (PMC4807087; doi:10.1371/journal.pmed.1001977)
Supplement: S1 Table — (DOCX) [file pmed.1001977.s011.docx]

**S1 Table. Literature search methodology and results, by database**

| **Database** | **Search terminology** | Results |
| --- | --- | --- |
| **Embase** | 1. exp influenza/ OR exp influenza virus/ OR exp influenza A/ OR exp influenza B/ OR exp influenza C/ OR exp influenza virus A / OR exp influenza virus B / OR exp influenza virus C / OR exp seasonal influenza/ OR influenza.mp OR viral etiology.mp  2. exp respiratory tract infection/ OR exp acute respiratory tract disease/  3. exp pneumonia/ OR exp virus pneumonia/  4. exp bronchiolitis/ OR exp viral bronchiolitis/ OR exp bronchitis/  5. exp flu like syndrome/ OR influenza-like illness.mp  6. ILI.mp OR SARI.mp OR ARI.mp OR ALRI.mp OR LRTI.mp OR CAP.mp  **7. 1 AND (2 OR 3 OR 4 OR 5 OR 6)**  8. Limit 7 to (human and yr=”1996-Current”) | 34349 |
| **Web of Science** | (TS= (Influenza) OR TS=(viral etiology))  AND  (TS=(Acute Respiratory Infections) OR TS=(respiratory diseases) OR TS=(respiratory illnesses) OR TS=(Pneumonia) OR TS=(influenza-like illness) OR TS=(lower respiratory tract infections) OR TS=(bronchiolitis) OR TS=(bronchitis))  Time span= 1996-2012 | 6018 |
| **Global Health** | 1. exp influenza/ OR exp influenza viruses/ OR exp Influenzavirus A/ OR exp Influenzavirus B/ OR exp Influenzavirus C/ OR influenza.mp OR viral etiology.mp  2. exp respiratory diseases/ or exp lower respiratory tract infections  3. exp pneumonia/  4. exp bronchiolitis/ OR exp bronchitis/  5. influenza-like illness.mp OR ILI.mp OR SARI.mp OR ARI.mp OR ALRI.mp OR LRTI.mp OR CAP.mp  **6. 1 AND (2 OR 3 OR 4 OR 5)**  7. Limit to yr=”1996-Current” | 4068 |
| **Pubmed** | 1. influenza, human[sh] OR influenza A virus[majr] OR influenza B virus[majr] OR influenza C virus[majr] OR influenza[all] OR viral etiology[all]  2. respiratory tract infections[sh] OR respiratory tract diseases[sh]  3. pneumonia[majr] OR pneumonia, viral[sh]  4. bronchiolitis[sh] OR bronchiolitis, viral[sh] OR bronchopneumonia[sh]  5. influenza-like illness[all]  6. acute respiratory[all] OR acute lower respiratory[all] OR lower respiratory[all]  7. ILI[all] OR SARI[all] OR ALRI[all] OR LRTI[all] OR CAP[all]  8**. 1 AND (2 OR 3 OR 4 OR 5 OR 6 OR 7)**  9. Limit 8 to ("1996/01/01"[PDAT] : "3000/12/31"[PDAT]) AND "humans"[MeSH Terms]) | 3596 |
| **CINAHL** | (TX influenza OR TX influenza virus OR TX viral etiology)  AND  (TX respiratory infections OR TX pneumonia OR TX influenza-like illness OR TX acute respiratory OR TX lower respiratory OR TX "ILI" OR TX "SARI" OR TX "ALRI" OR TX "ARI" OR TX "LRTI" OR TX "CAP")  Limiters: 1996-2012; human | 630 |
| **LILACS** | (Influenza)  AND  (respiratory disease OR respiratory infection OR OR pneumonia) | 275 |
| **WHOLIS** | (influenza OR influenza virus OR viral etiology)  AND  (respiratory infection OR respiratory disease OR respiratory illness OR acute respiratory OR lower respiratory OR pneumonia OR bronchiolitis OR bronchitis OR SARI OR ILI OR ARI OR ALRI OR LRTI OR CAP)  Publication year: 1996-2012 | 10 |
| **IndMed** | (Influenza)  AND  (respiratory disease OR respiratory infection OR OR pneumonia) | 4 |
| **CNKI** | 甲流 or 流感 or 病毒病原学 (influenza group keywords) And 呼吸系统感染 (respiratory infection)  甲流 or 流感 or 病毒病原学(influenza group keywords )And 呼吸系统疾病 ( another name of respiratory infection)  甲流 or 流感 or 病毒病原学 (influenza group keywords) And 肺炎 or下支气管炎 (pneumonia or bronchiolitis )  甲流 or 流感 or 病毒病原学 (influenza group keywords) And 肺炎 or 上支气管炎 (pneumonia or bronchitis )  甲流 or 流感 or 病毒病原学 (influenza group keywords) And 流感病样病例 (ILI Chinesename) or ILI  甲流 or 流感 or 病毒病原学 (influenza group keywords) And 急性呼吸道感染( sari chinese name) or SARI  甲流 or 流感 or 病毒病原学 (influenza group keywords) And ARI  甲流 or 流感 or 病毒病原学 (influenza group keywords) And 急性下呼吸道感染 (LRTI Chinese name) or LRTI  甲流 or 流感 or 病毒病原学 (influenza group keywords) And 急性上呼吸道感染 (LRTI Chinese name) or LRTI  甲流 or 流感 or 病毒病原学 (influenza group keywords) And 非典型肺炎 (SARS Chinese name) or SARS  甲流 or 流感 or 病毒病原学 (influenza group keywords) And 社区活动感染 (CAP Chinese name) or CAP | 883 |
